# Extracellular vesicle biomarkers in circulation for colorectal cancer detection: a systematic review and meta-analysis

**DOI:** 10.1186/s12885-024-12312-8

**Published:** 2024-05-22

**Authors:** Xianquan Shi, Xinyu Zhao, Jinru Xue, Erna Jia

**Affiliations:** 1 Department of Gastroenterology, China-Japan Union Hospital of Jilin University, Changchun, China; 2 Department of Thoracic Surgery, China-Japan Union Hospital of Jilin University, Changchun, China; 3 Department of Ultrasound, Beijing Friendship Hospital, Capital Medical University, Beijing, China; 4 Clinical Epidemiology & EBM Unit, Beijing Friendship Hospital, Capital Medical University, National Clinical Research Center for Digestive Diseases, Beijing, China

**Keywords:** Extracellular vesicles, Diagnosis, Circulation, Biomarker, Colorectal cancer

## Abstract

We provided an overview which evaluated the diagnostic performance of circulation EV biomarkers for CRC from PubMed, Medline, and Web of Science until 21 August 2022.Weidentified 48 studies that involved 7727 participants and evaluated 162 plasma/serum individual EV biomarkers including 117 RNAs and 45 proteins, as well as 45 EV biomarker panels for CRC detection. 12 studies evaluated the diagnostic performance of EV biomarkers for early CRC. The summarized sensitivity, specificity, and AUC value of individual EV RNAs and EV RNA panels were 76%, 75%, 0.87 and 82%, 79% and 0.90, respectively. Meanwhile, those of individual EV proteins and EV protein panels were 85%, 84%, 0.92 and 87%, 83%, 0.92, respectively. These results indicated that EV biomarker panels revealed superior diagnostic performance than the corresponding individual biomarkers. In early CRC, EV biomarkers showed available diagnostic value with the sensitivity, specificity, and AUC value of 80%, 75%, and 0.89.In subgroup analyses, EV miRNAs and LncRNAs held similar diagnostic value with the sensitivity, specificity and AUC value of 75%, 78%, 0.90 and 79%, 72%, 0.83, which was highly consistent with the whole EV RNAs. Significantly, the diagnostic values of EV miRNAs in plasma were marginally higher than those based on serum. In detail, the sensitivity, specificity, and AUC values were 79%, 81%, and 0.92 in plasma, as well as 74%, 77%, and 0.88 in serum, respectively. Therefore, circulation EV biomarkers could be considered as a promising biomarker for the early detection of CRC.

**Supplementary Information:**

The online version contains supplementary material available at 10.1186/s12885-024-12312-8.

## Introduction

Colorectal cancer (CRC) is the third most common cancer and the second leading cause of cancer death worldwide [1]. More than 1.9 million new cases and 935,000 deaths have occurred in the United States in 2020 [1].In people younger than 50 years old the CRC incidence rate increases by 1.5% per year from 2014 to 2018 [2]. The 5-year survival rate for advanced CRC is less than 20%, whereas the 5-year relative survival rate for early stage CRC can reach 90.9% [3]. Thus, the US preventive services task force has recommend major expansions of the routine screening for CRC in 2021 [4].

Recommended screening instruments for the risk population are fecal occult blood test and colonoscopy. Fecal occult blood test is more affordable, less invasive, cost-effective, and more specificity for advanced CRC, but its sensitivity is limited both for early and advanced CRC [5, 6].Although endoscopy has a higher sensitivity and specificity for CRC diagnosis, it is expensive, time-consuming, and invasive, which also increases the psychological and social burden on patients [7].Carcinoembryonic antigen (CEA) is another noninvasive method for diagnosing CRC, but it has lower sensitivity and is always significantly elevated in benign diseases [8]. Therefore, anticipating novel noninvasive biomarkers with powerful diagnostic efficiency as screening strategies for early detection of CRC is critical.

Extracellular vesicles (EVs), which are membrane-bound particles secreted by nearly all cells, exist in various body fluids and contain RNA, DNA, protein, and lipids. It is well known that EVs can reflect the parent cell of origin, transmit information between cells, as well as participate in their physiological and pathological processes. Recently, EVs RNAs and proteins as valuable noninvasive biomarkers have garnered considerable interest for several cancer screening and diagnosis including pancreatic cancer, prostate cancer, gastric cancer, and CRC [9, 10]. However, whether EV RNAs or EV proteins are benefit to detection and screening early cancers is inconclusive. Both plasma and serum EV biomarkers have been demonstrated as valuable biomarkers for cancers in numerous studies, however whether plasma or serum can be as an ideal source of circulation EVs without affecting the experimental results is still unclear. The aim of this study was to summarize the diagnostic performance of circulation EV RNAs and EV proteins for CRC detection and to understand the diagnostic value of EV miRNAs in different circulation specimens.

## Methods

The present review and meta-analysis followed a preferred protocol and the PRISMA guidelines [11].

### Selection of studies

We searched PubMed, Medline, and Web of science databases for literature with the following MeSH terms up to 21 August 2022: ((Colorectal OR colo* OR rect*) AND (cancer OR carcinoma OR neoplasm OR tumor OR malignancy OR adenocarcinoma OR adenoma)) AND (detection OR diagnosis OR biomarker OR marker OR sensitivity OR specificity OR area under the curve) AND (exosome OR Extracellular Vesicles OR exosomal OR membrane vesicles OR intracellular multivesicular endosomes). The search was restricted to studies evaluating circulation EV biomarkers for CRC detection. Duplicates were deleted.

Non-English articles, non-original articles, non-human studies, not-related CRC articles, and articles not relevant to the topic were all excluded. Then, two investigators (Jinru Xue and Na Ren) independently reviewed all potentially relevant studies, the following studies were included: (1) studies that identified EV biomarkers for diagnosing CRC in serum, plasma, blood, or peripheral blood; (2) CRC patients were diagnosed depending on the cytological or histological examination; (3) studies reported the diagnostic value of EV biomarkers for CRC including sensitivity, specificity, area under the curve (AUC), or receiver operator characteristic (ROC) curve. Discrepancies were resolved through discussion.

### Data abstraction and assessment of methodological study

Pre-designed data collection tables were used and the two investigators extracted available information from eligible studies using the tables. The key information included first author, year of publication, country, population characteristics (including sample size, mean age, and gender distribution), types of blood-based specimens, CRC stage, population composition of control groups, names or panels of target biomarkers, detection methods of target biomarkers, preparation approaches of EVs, sensitivity, specificity, and AUC. The risk of bias and application for eligible studies were assessed using the Quality Assessment of Diagnostic Accuracy Studies 2 (QUADAS-2) check list by Review Manager 5.3 [12]. A funnel plot was used to assess the potential of publication bias, and we used R software (version 3.5.3, R Foundation, Vienna, Austria) to perform egger’s test to assess funnel plot symmetry [13].

### Statistical analysis

The mean age and sex distribution were calculated using raw data by R software if these two data were not reported in eligible studies. We also explored the values of sensitivity and specificity based on ROC curves using OriginPro software (version 9.0) according to the maximum Youden’s index, if these two diagnostic indicators were not reported.

We summarized the sensitivity, specificity, and AUC value of EV biomarkers among eligible studies with relevant data Using metaDisc software (version 1.4) by the random-effect model (DerSimonian-Laird method). The control groups contained healthy controls and/or benign diseases, we studied the healthy controls if the relevant data was available, or we studied them as a whole. The heterogeneity across studies was assessed by Cocharan’s Q test and the inconsistency index (*I*^2^ value), with *P*<0.05 or *I*^2^>50% as statistically significant heterogeneity. We performed subgroup analysis to summarize the sensitivity, specificity, and AUC value of individual EV microRNAs (miRNAs), and individual EV long non-coding RNAs (LncRNAs) for CRC diagnosis. We also observed the summarized diagnostic value of the individual EV miRNAs in serum and plasma for CRC diagnosis, respectively. Finally, we conducted sensitivity analysis to assess the diagnostic value of individual EV miRNAs detected by qPCR for CRC.

## Results

### Results of the search

**Fig. 1:**
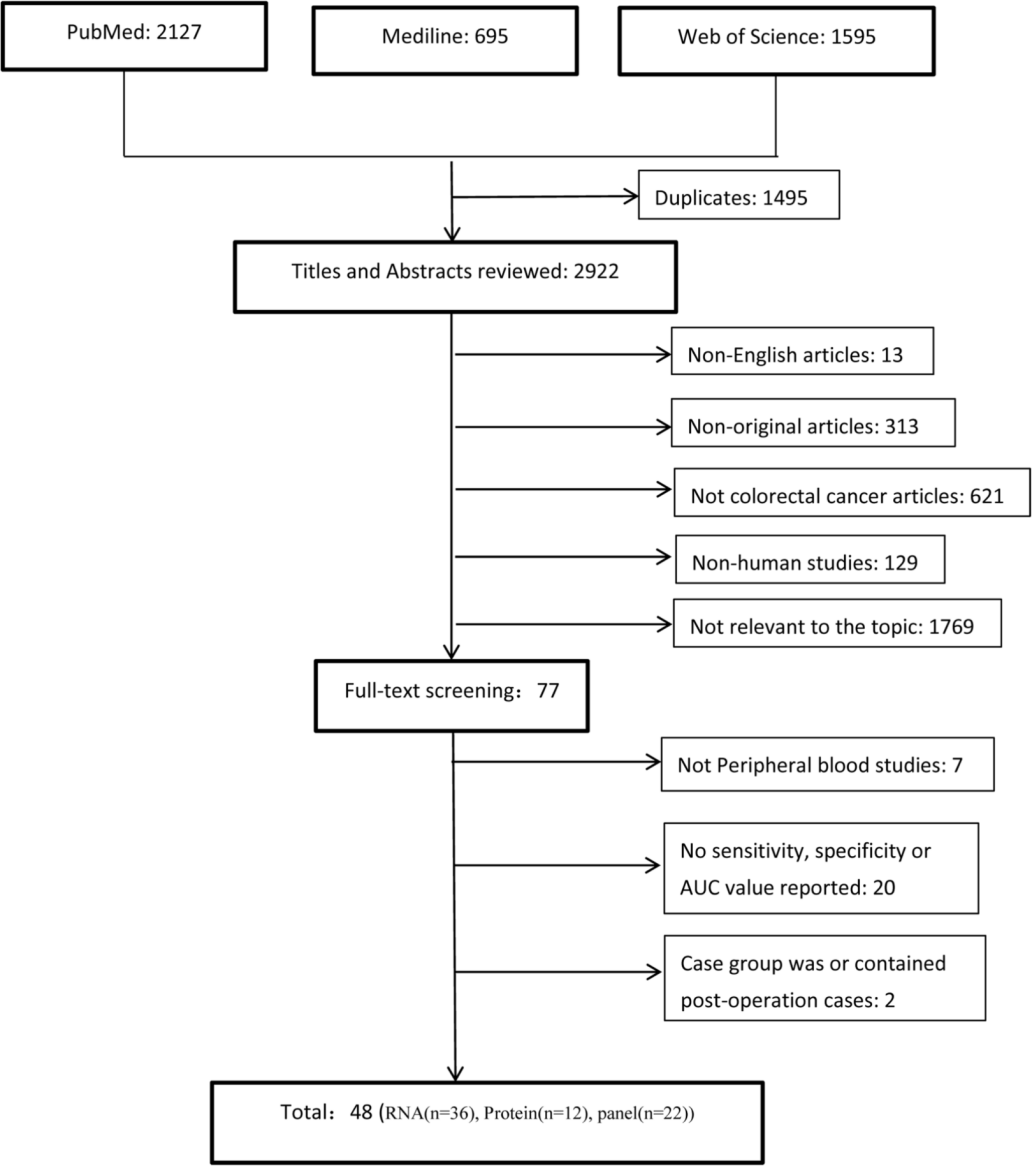
The flow chart of the study

We identified 4417 studies from the initial search of databases and removed 1495 duplicate studies. We screened the titles and abstracts of 2922 studies and retrieved 77 full-text studies for eligibility assessment. 29 studies were excluded: the specimens of 7 studies were not peripheral blood;20 studies had no sensitivity, specificity, AUC value, or ROC curve; the control group of one study was all post-operation CRC patients, and in another study the control group contained several post-operation CRC patients. Finally, we identified 48 eligible studies for qualitative and quantitative analysis. The flow chart was shown in Fig. 1.

### Studies characteristics

The 48 eligible studies were all case-control researches with 4369 CRC and 3358 controls [14–61] 0.39 studies were carried out in Asia, eight were in Europe, and one in North America. The mean sample size of CRC groups was 62 (range from 6 to 410), and the mean sample size of control groups was 56 (range from 5 to 100). Table S1-S3 elaborated the detail information of the eligible studies, including mean age, sex distribution, number of cases and controls, detection methods, and CRC clinical stage. Thirty-seven studies reported the diagnostic value of individual EV RNAs (miRNAs in twenty-three studies, lncRNAs in eight studies, circular RNAs (cirRNAs) in 4studies, messenger RNA (mRNAs) in two studies), 8 of which conducted validation tests (one conducted external validation test); nine studies reported individual EV proteins, 2 of which conducted independent validation tests; 22 studies reported EV biomarker panels, five of which conducted validation tests (containing three external validation tests); and twelve studies reported the diagnostic performance of EV biomarkers for early stage (stage I and II) CRC, 5 of which conducted validation tests containing 2 external validation tests.

Ultracentrifugation (UC) is currently recommended and the most widely used method for EV extraction and separation. In the present review, 22 studies used UC for EV extraction, 21 studies used different commercial kits, one used a size-based isolation technique, one used immunoaffinity chromatography, one used a Two-Phase Polymer System, and two did not report the extraction and isolation methods (Table S4).

### Methodological quality of included studies

The results of the methodological quality of the included studies were summarized in Fig. S1. In total, 23 studies had unclear risk of patient selection bias because of non-consecutive or non-random patient selection. 6 studies had unclear concern of patient selection because gender distribution and mean age were not reported. All 48 studies were of low risk of bias for index test, reference standard and flow and timing. All 48 studies were of low concern for application with regard to the index test and the reference standard. The funnel plot showed reasonably symmetrical, and Egger’s test revealed no evidence of publication bias (*P* = 0.23, Fig. S2).

### Diagnostic efficiency

A total of 117 individual EV RNAs (59 contained in panels) with significantly potential diagnostic capability for CRC were reported in 36 eligible studies, and both the sensitivity and specificity of 35RNAs exceeded 80%. Ten RNAs were reported in more than one study (Table 1), eight of which also appeared in panels (Table S5). The most frequently reported RNA was miR-21 in five studies, with the sensitivity ranging from 60 to 95% and the specificity ranging from 50 to 100%, respectively. In two studies, both the sensitivity and specificity of miR-21 exceeded 90%, and the specificity even reached 100% [35, 44].Shi Y et al. discovered and validated four miRNAs including miR-126, miR-1290, miR-23a, and miR-940withexcellent diagnostic efficiency and AUC value greater than 0.85; Additionally, authors observed and validated that miR-126, miR-1290, miR-23a, and miR-940 had respectably diagnostic performance with AUC value greater than 0.80 for CRC with stage I [21]. Among 36 eligible studies, 11 studies evaluated diagnostic performance of RNAs for early stage (stage I and II) CRC. CircRNAs seemed to present greater diagnostic efficiency than other RNAs. In an independent validation test, Pan B et al. discovered circ-0004771 was significantly up-regulated in serum of CRC patients with stage I-IIb compared to healthy control, with sensitivity of 81% and specificity of 80% [42]. Validation test in Xie Y et al’s study showed circ-PNN was clearly up-regulated in CRC patients with stage I and II, the sensitivity, specificity, and AUC value were 92%, 69%, and 0.85, respectively [30].

**Table 1:** Summary of studies reporting significant associations of RNAs more than once in colorectal cancer

RNAs	(61)	(60)	(58)	(55)	(54)	(53)	(52)	(51)	(48)	(47)	(50)	(45)	(44)	(43)	(42)	(40)	(39)	(37)	(35)	(34)	(33)	(31)	(30)	(29)	(28)	(36)	(26)	(24)	(22)	(21)	(19)	(18)	(17)	(16)	(15)	(14)	Number of studies
miR-21	↑○					↑△							↓○						↑○									↑△									5
miR-150	↑○																↓○	↓○																			3
miR-122						△															↑○																2
miR-139-3p													↑△							↓○																	2
miR-19a						↑△							↓○																								2
miR-19b													↓○						↑○																		2
miR-23a	↑○																													↑△							2
miR-381			△																																↓△		2
miR-425						↑△							↓○																								2
miR-92								↑△											↑○																		2

○ represents RNAs which have only been analyzed individually and not as part of a miRNA panel; △ represents RNAs which are part of a panel; ↑ represents up-regulation; ↓ represents down-regulation

A total of 45 individual EV proteins (21 contained in panels) with diagnostic value for CRC were reported in 12 studies, both the sensitivity and specificity of 23 proteins exceeded 80%. Four proteins were reported more than once, all of which were also reported in panels (Table S6). EpCAM and CD63 were most frequently reported in 3 studies. Several EV proteins presented excellent diagnostic value for CRC detection. For example, Zheng X et al. [27]discovered that the sensitivity, specificity, and AUC value of FGA for CRC detection were 100%, 100%, and 1.00, respectively; Shiromizu T et al [56]separately investigated the diagnostic value of 22 EV proteins for CRC patients with stage I and II. In the external validation test, all 22 proteins could distinguish CRC patients form healthy controls. For patients with stage I, the AUC value of 18 proteins was greater than 0.80, and the AUC value of ANXA11, ANXA3, ANXA4, TFRC, GLUT-1, CD88, MMP9, CEACAM8, ANXA5, OLFM4, and LCN2 were greater than 0.90.

45 EV biomarker panels with diagnostic performance for CRC were derived among 21 studies, seven of which was verified in validation test, and more than half of the panels (29 panels) with both the sensitivity and specificity exceeded 80%. Wei P et al. discovered that CD63combined with EpCAM had 100% sensitivity and 100% specificity, while CD63 combined with CD9 had 93%sensitivity and 96% specificity, respectively. Shiromizu T reported 13 EV protein panels for distinguishing CRC patients with stage II from healthy controls; the results demonstrated high diagnostic power, with AUC values all exceeding 0.80 [56]. 4 RNA panels performed highest sensitivity for diagnosing CRC, with all of them reaching 100% [36, 52].One miRNA panel comprised of miR-92a and miR-141 showed95% sensitivity and 100% specificity [17]. In general, EV biomarker panels outperformed individual EV biomarkers for CRC diagnosis.

### Results of meta-analysis

**Fig. 2:**
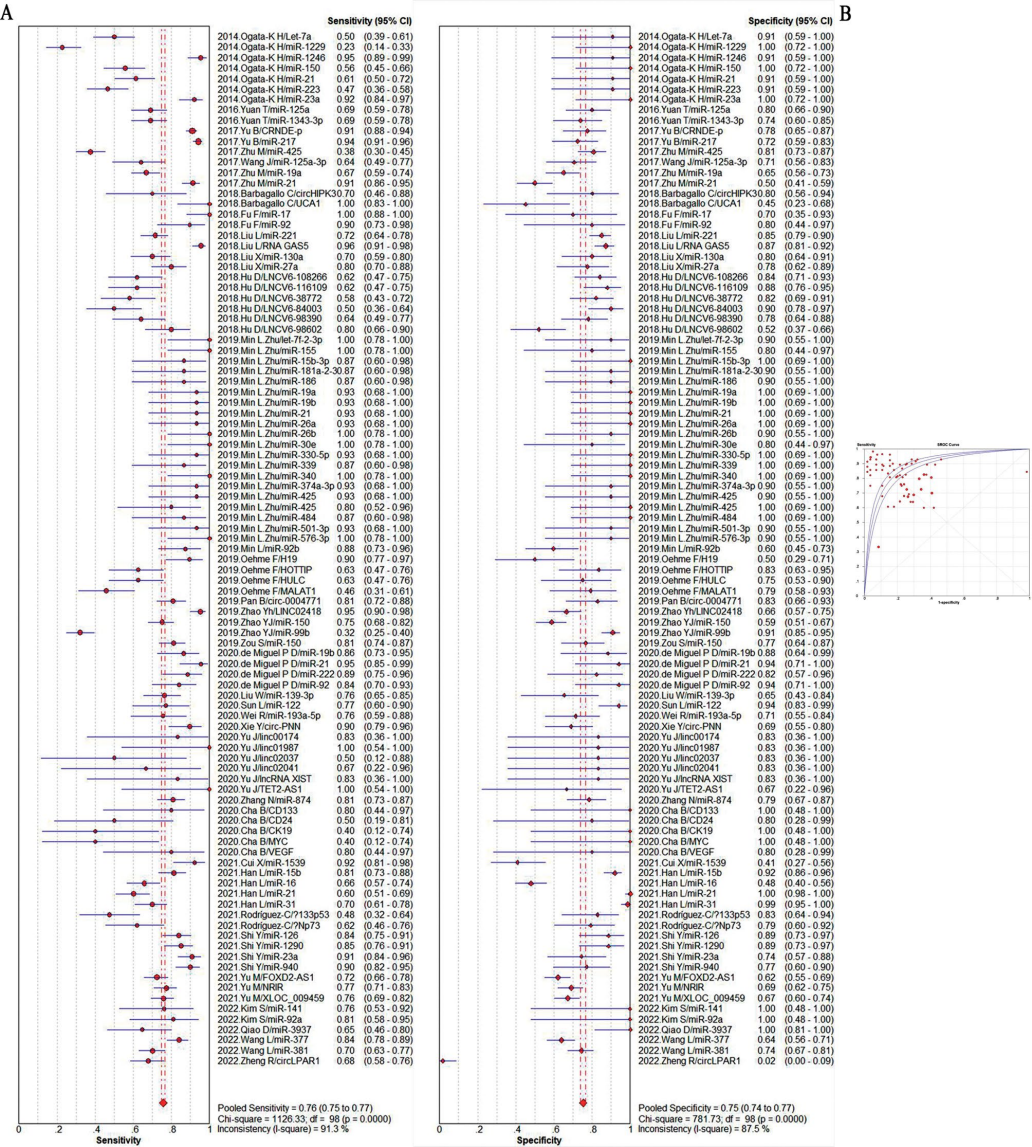
The summary of diagnostic performance of EV RNAs for colorectal cancer including (**A**) forest plot, (**B**) ROC curve

**Fig. 3:**
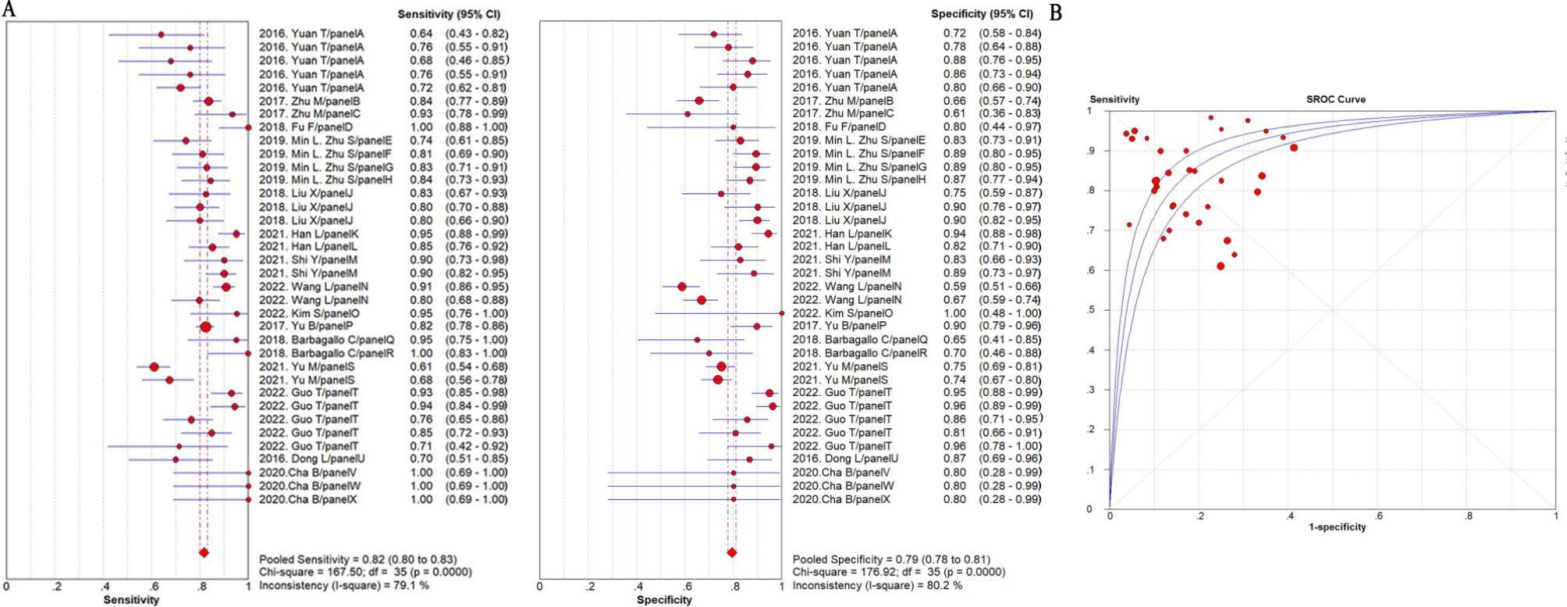
The summary of diagnostic performance of EV RNA panels for colorectal cancer including (**A**) forest plot, (**B**) ROC curve

**Fig. 4:**
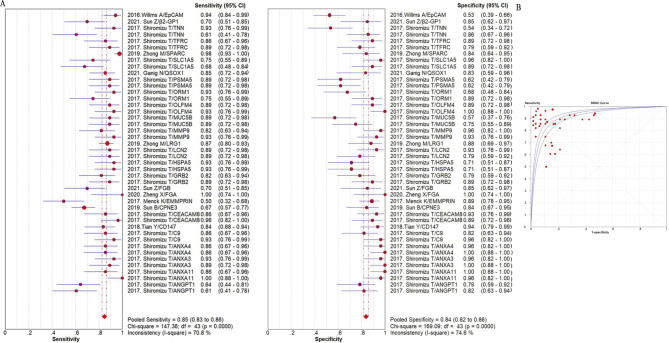
The summary of diagnostic performance of EV proteins for colorectal cancer including (**A**) forest plot, (**B**) ROC curve

**Fig. 5:**
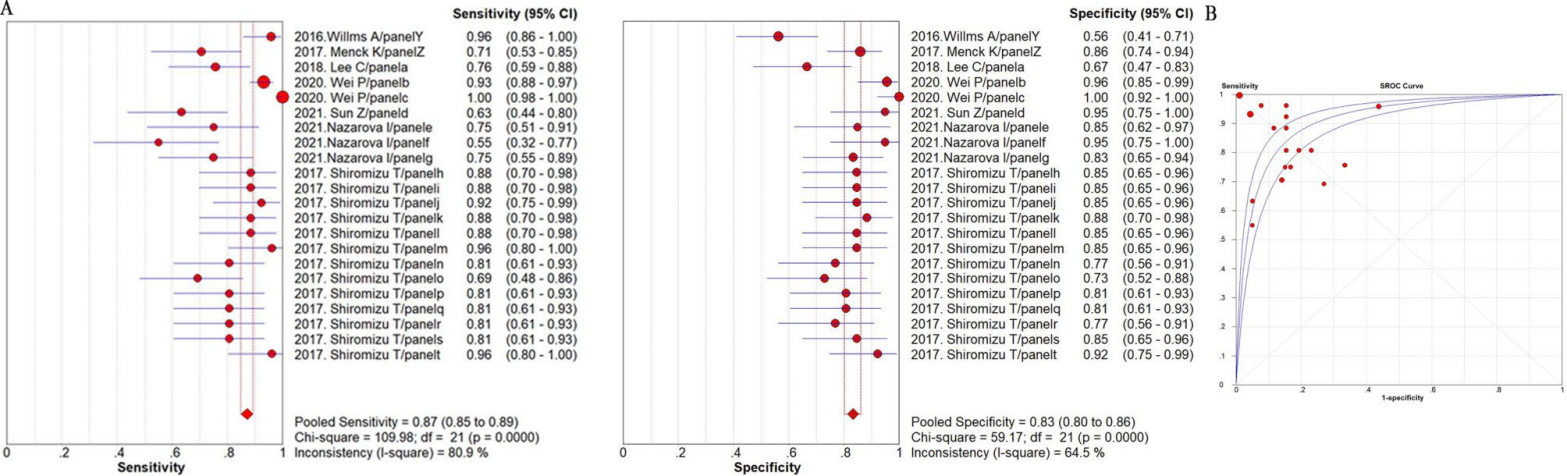
The summary of diagnostic performance of EV protein panels for colorectal cancer including (**A**) forest plot, (**B**) ROC curve

**Fig. 6:**
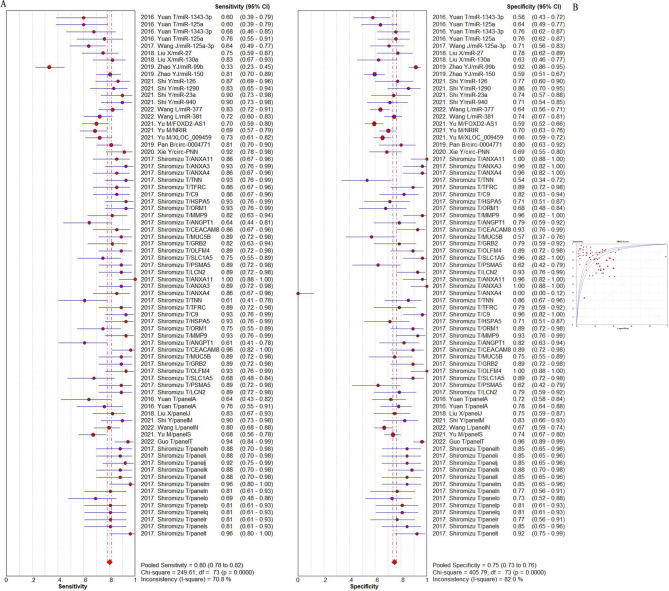
The summary of diagnostic performance of EV biomarker for colorectal cancer with stage I-II including (**A**) forest plot, (**B**) ROC curve

The summarized sensitivity, specificity, and AUC value of EV RNAs for diagnosing CRC were 76%, 75% and 0.86(Fig. 2); and 82%, 79%, and 0.90for RNA panels, (Fig. 3). The summarize sensitivity, specificity, and AUC value of EV proteins for diagnosing CRC were85%, 84% and 0.92 (Fig. 4); 87%, 83%, and 0.92 for protein panels (Fig. 5). Overall, EV biomarker panels revealed greater diagnostic efficiency than the corresponding individual EV biomarkers for CRC. CRC stage subgroup analysis carried out in twelve studies. We summarized the diagnostic value of the EV biomarkers for CRC patients with stage I-II, the sum of the sensitivity, specificity, and AUC value were 80%, 75%, and 0.89, which indicated their relative good diagnostic performance (Fig. 6).

**Fig. 7:**
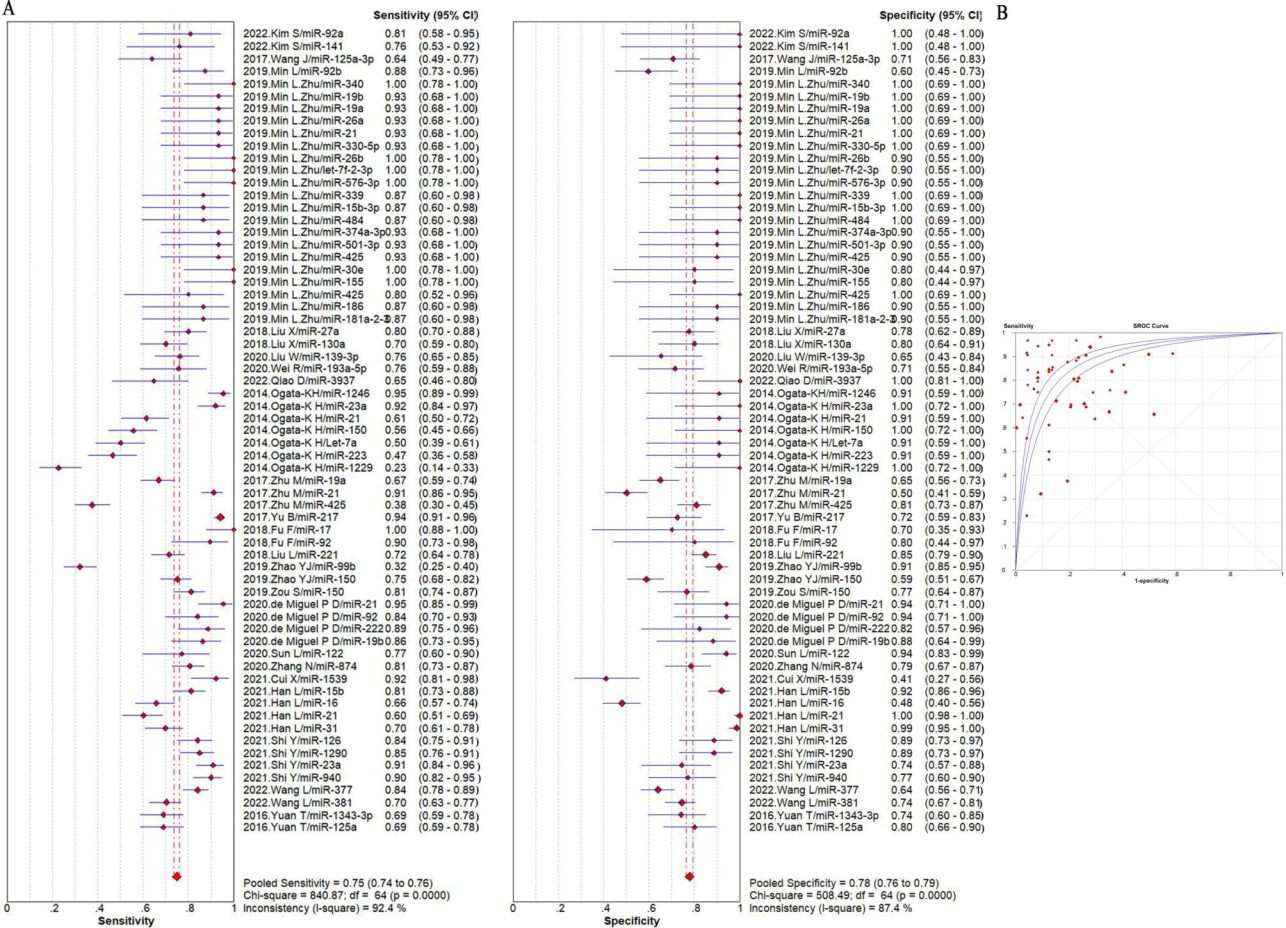
The summary of diagnostic performance of EV miRNAs for colorectal cancer including (**A**) forest plot, (**B**) ROC curve

**Fig. 8:**
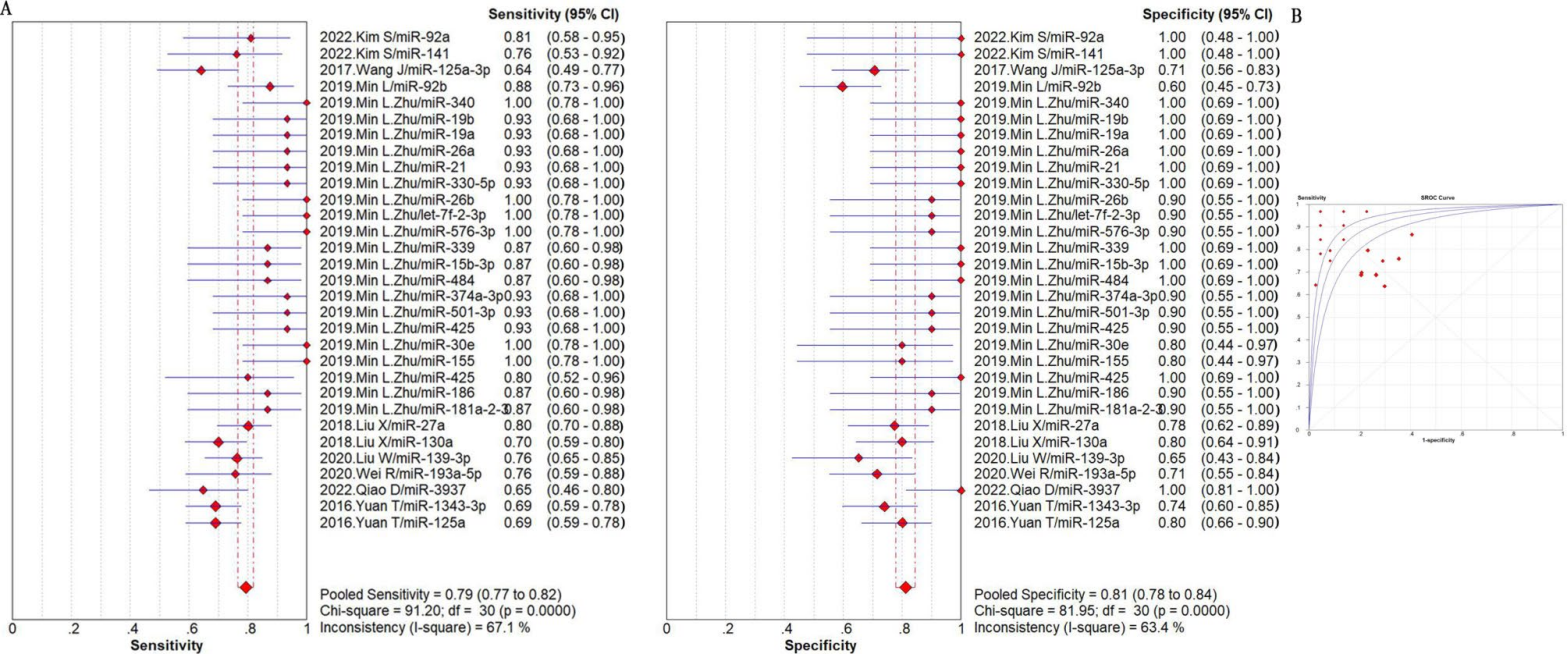
The summary of diagnostic performance of EV miRNAs for colorectal cancer in plasma subgroup including (**A**) forest plot, (**B**) ROC curve

**Fig. 9:**
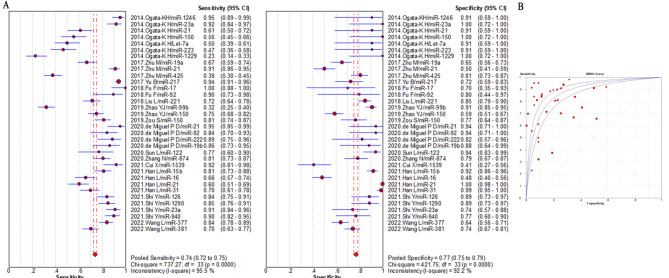
The summary of diagnostic performance of EV miRNAs for colorectal cancer in serum subgroup including (**A**) forest plot, (**B**) ROC curve

To explore the diagnostic advantage of EV RNAs, we performed subgroup analysis based on miRNA and LncRNAs. The sum of the sensitivity, specificity, and AUC value of EV miRNAs were75%, 78%, and 0.90(Fig. 7); the sum of the sensitivity, specificity, and AUC value of EV LncRNAs were79%, 72%, and 0.83 (Fig. S3). The diagnostic value of EV miRNAs and EV LncRNAs were found to be consistent with the whole EV RNAs. Subgroup analysis were also used to summarize the diagnostic value of EV miRNAs in plasma and in serum. It was easily found that the diagnostic value of EV miRNAs in plasma was slightly higher than that in serum. In detail, the summarized sensitivity, specificity, and AUC value were 79%, 81%, and 0.92 (Fig. 8), and 74%, 77%, and 0.88 (Fig. 9), respectively. Sensitivity analysis was then used to assess the diagnostic performance of EV miRNAs detected by qPCR. The result demonstrated that the sum of the sensitivity, specificity, and AUC value were75%, 78%, and 0.90, respectively, which was similar with the whole miRNAs (Fig. S4).

### Regulation direction of EV RNAs

The majority of RNAs were reported in 1 study and the dys-regulation direction of these RNAs was consistently up-regulated. 10 miRNAs were reported in at least 2 studies, and 7 of them had contradictory directions (Table 1). MiR-21, the most frequently reported RNA, was up-regulated in 4 studies, down-regulated in 1 study. MiR-150was down-regulated in 2 studies and up-regulated in 1 study.

## Discussion

Our review concentrated on the diagnostic performance of plasma/serum EV RNAs and EV proteins for CRC. 48 studies met the inclusion criteria for evaluating the diagnostic performance of 117 EV RNAs and 45 EV proteins based on serum/plasma for detection CRC from 2014 to 2022. The control groups of most included studies were healthy people while only 4 studies chose noncancerous populations as control groups including adenoma, benign intestinal diseases, as well as vascular diseases.22 studies integrated individual RNAs or proteins into panels and found that the diagnostic performance of panels generally outperformed that of individual RNAs or proteins.12 studies focused on the diagnostic performance of EV biomarkers for CRC patients with stage I-II and further demonstrated their powerful diagnostic efficiency with an AUC value of 0.89.Although promising, well-designed prospective diagnostic accuracy studies are highly required owing to the fact that all of the included studies were case-control tests.

EVs derives from the original of cells contained similar nucleic acids and proteins, which played a crucial role in the communication between cancer cells themselves and between the cancer and cancer microenvironment. MiRNAs, LncRNAs, mRNAs, and circRNAs belonged to noncoding RNAs, which could not encode protein and perform their biological functions at the RNA levels. RNAs could modulate several signaling pathways in colorectal cancer cell proliferation, apoptosis, and migration. Eoxsome miR-25-3p promoted colorectal cancer development by inducing vascular permeability and angiogenesis [62].MiR-590-5p was upregulated in the CRC tissues compared with normal tissues, which inhibited CRC angiogenesis mainly by affecting NR-90/VEGF-A, reducing the enhanced migration ability of cancer cells [63]. Exosome LcnRNA-UCA1could promote CRC cell proliferation via the miR-143/MYO6 axis.LcnRNA-UCA1 could be transmitted into CRC cells, resulting in the increased expression of MYO6 by sponging with miR-143 and promoting the malignancy of CRC [64].CircLONP2 could modulate the maturation and exosomal dissemination of miR-17 to enhance the invasion and metastasis of CRC [65]. Circ-IFT80 contributed to the tumorigenesis of CRC via regulating miR-296-5p/MSI1 axis [66]. EV proteins could reflect their subcellular origin and the donor cell type, directing their targeting and capture by recipient cells. For example, MUC1/CA153 could promote tumor invasion when expressed in its highly-glycosylated isoform [67]. Therefore, protein profiling of EVs was also indispensable for CRC diagnosis.

EVs could facilitate intercellular communication by transferring genetic information via RNAs including miRNAs, mRNAs, and LncRNAs [68]. RNAs could directly represent the expression level of specific genes, as well as mediate cancer development and metastasis [69]. EV RNAs could protect from RNase-mediated degradation and stably existed in plasma and serum [70].Therefore, circulation EV RNAs were considered as novel noninvasive biomarkers for CRC, and numerous studies indicated that EV RNAs could differentiate CRC patients from noncancerous and healthy controls. Similar to previous findings, we observed a large number of EV RNAs with available diagnostic performance for CRC, but the overlap rates of these RNAs were low. Among all these RNAs, miRNAs and lncRNAS were studied most extensively, both these two RNAs could directly regulate the gene expression at epigenetic, transcriptional, and posttranscriptional level. The expression levels of miRNAs and LncRNAs contained in EVs were abundant while their function were well studied in various pathological and phrsiological processes [71]. Where the levels of circRNAs in EVs might be modulated by changes in associated miRNA levels in donor cells, and circRNAs serves as miRNA and protein sponges [72, 73], the function of which existing in EVs still lacked evidence.MiR-21 was the most frequently reported and the regulation direction of most of them was upregulated, indicating which might be a promising EV miRNA for CRC diagnosis. As the first oncomiRs, MiR-21was also found upregulated and studied as a promising diagnostic and prognostic biomarker for several other cancers [74], whether miR-21 could be as a CRC-specific diagnostic biomarker needed analysis. The regulation direction of the remaining repeatedly reported miRNAs, except miR-92, were almost contradictory. The inconsistencies in these studies needed more repetition results to demonstrate. EpCAM, CD63, CD9, and CD147 were repeatedly recognized as positive protein in EVs in CRC, the 4proteins were all tetraspanins (also termed 4-transmembrane cross-linked proteins) and were indicated to facilitate the entry of specific cargos into EVs [75].Thus, these 4 proteins could be used as a biomarker panel, which was also specificity for CRC, to improve the diagnostic efficiency for CRC. Taylor et al. suggested that using cancer-specific EVs, such as EpCAM-positive or GPC1-positive EVs, could help overcome the limitation and improve the diagnostic efficiency for CRC [76, 77].In the current review, EV proteins revealed superior diagnostic performance for CRC, with summarized diagnostic values that were higher than EV RNAs. Thus, combining EV RNAs and proteins might improve the sensitivity and specificity for CRC diagnosis. However, large population-based cross-sectional studies were still needed to identify optimal EV RNA and protein panels that could be used in clinical care to diagnose early stage CRC.

Blood was the richest source of EVs, as well as the composition profiles of plasma and serum were similar. Plasma and serum were both used as the potential sources of circulation EVs, though plasma was more commonly used [78].In this study, we conducted subgroup analysis to separately summarize the diagnostic value of EV miRNAs in serum and plasma for CRC. Similar with the previous studies, the findings indicated that the diagnostic value of EV miRNAs in plasma was slightly higher than that in serum, implying that plasma was more suitable as a source of circulation EVs biomarkers for diagnosing CRC. It was well known that platelet could release a portion of EVs especially when activated [79]. During the process of serum collection, blood coagulation could activate platelet. Consequently, serum EVs were highly contaminated by platelet-derived EVs, which might qualitatively and quantitatively alter EV profiles when serum used as a source of circulation EVs [57, 80–82]. Several anticoagulants were used during the plasma collection, including EDTA, citrate, and heparin. Citrate or EDTA could decrease or eliminate EVs in plasma by inducing EVs to bind to platelets or other formed elements [83]. Palviainen et al. also detected different particle numbers and proteins of EVs in plasma collected using EDTA, citrate, and acid citrate [81]. But Zhang X et al. demonstrated the numbers and diameters of EVs exhibited no differences in plasma collected using EDTA, citrate, and heparin [80]. It has been reported that calcium chelators, such as EDTA and citrate, but not heparin, promote the association of EVs and platelets, and lower the apparent count of EV particle in plasma [83]. The use of EDTA, citrate, and acid citrate dextrose (ACD) results in differences in particle number and protein profiles of plasma EVs [81]. One recent side-by-side study reported that CD9+/CD41a + EVs are released during blood collection or released in vitro in the collection tube by comparing different anticoagulants, and that ECTA-plasma contains more residual platelets and CD9 + EVs than ACD-plasma and serum and the differences in CD9 + vesicles might therefore be at least partly due to post-collection activation of platelets in EDTA tubes [84]. However, another study using blood samples form mice demonstrated the numbers and diameters of EVs in plasma collected using EDTA, citrate, and heparin had no differences [80]. Therefore, more research was highly needed to determine whether the use of anticoagulants showed an effect on the EV biomarkers in plasma.

Mircrovesicles (MVs) with a size of 50–1000 nm and exosomes with a size of 40–100 nm were collectively called EVs used in the cancer biomarker research. These two types of vesicles differed not only in size but also in origin. MVs were directly released form cell membranes, whereas exosomes were intracellular in origin. Although the biomolecules including DNA, RNA, lipid and proteins contained in EVs were highly similar, their concentrations differed, as well as exosomes were the richest reservoir for mRNAs and lncRNAs [60, 85, 86].Thereby, exosomes might be ideal candidate RNA carriers when using RNAs to diagnose CRC. In this review, several studies used EVs RNAs as biomarkers for CRC, which could increase the heterogeneity of summarized sensitivity, specificity, and AUC values of subgroup analysis. Owing to the number of included studies only focused on miRNAs, mRNAs, or LcnRNAs in exosomes or MVs were limited, we did not conduct subgroup analysis to demonstrate the aforementioned issue. This issue must be resolved in the future for further application of these noninvasive biomarkers in daily clinical settings.

EVs were heterogeneous in size and count, making isolation and separation more difficult. Efficient extraction of EVs and development of a direct quantification method were major issues of circulation biomarkers. Recently, UC became the most widely used and the recommended method for EV isolation and separation. However, there was no uniform protocol standardization step in the centrifugation time, centrifugal force, rotor type, or parameters that influenced the purity and yield of EVs [87, 88]. In the current systematic review, 22 studies used UC to isolate EVs, with varying centrifugal times and numbers, which might highly affect the purity and concentration of target EVs. In addition, UC was not conducive to clinical application due to its time consumption, high cost, structural damage, aggregation into blocks, co-sedimentation, and lipoprotein co-separation [89, 90]. Size-based isolation techniques, immunoaffinity charomatography, and other new isolation techniques were also used for EV extraction, which might be suitable for extracting EVs from plasma and serum, but there were limited number of studies on these techniques [23, 36, 51]. Size-based isolation techniques separated molecules by virtue of their size, where molecules larger than the pores of the stationary phase pass through the column faster by avoiding entering the pores while smaller molecules diffuse into the pores and have longer retention times [91]. The advantages include preserving the structural integrity of EVs, low infrastructural demand; and the main disadvantage is the co-isolation of other components of similar sizes, such as lipoproteis [92, 93]. The immunoaffinity-based method enriches EVs expressing specific antibody-recognized proteins, only a subset of all EVs may be captured, which can result in a low yield and high quality EV isolation [94, 95]. Polymer-system based EVs isolation method strongly combines with water molecules while less soluble components like EVs precipitate, which takes the shortest time but results high level of contamination [96]. Most commercially EV isolation kits based on polymer-system base enrichment, such as Exoquick and Total Exosome Isoltaion Kit. EVs are emerging as a potential diagnostic and therapeutic tool. To achieve this diagnostic potential in clinical applications, fast and standardized EVs isolation method with small quantities of biosamples is essential, which cannot be achieved by using the conventional UC method. Although recent reports showed significantly reduced enrichment duration, for example, down to less than 1 h, using small amounts of biosamples, for example, less than 100 µl. With the development of the novel EVs isolation methods, applying this method in conjunction with other techniques might result in higher yield and purity and will be an essential contributor for EVs to be used in the clinical field.

## Conclusion

Circulation EV RNAs and relative proteins appeared to reveal great promise as novel noninvasive biomarkers for CRC detection in its early stage. Lots of scientific evidence demonstrated plasma/serum EV RNAs and proteins in cancer diagnosis, as well as the functional roles of these molecules contained in EVs in cancer development and metastatic, however verifying these EV RNAs and proteins was still critical. Meanwhile, standardization of methodology and specimen identification could reduce the bias in the diagnostic performance of EV biomarkers and aid in the clinical feasibility of EV RNAs and proteins for CRC diagnosis. Our systematic review thereby indicated that circulation EV biomarkers could be considered as a promising biomarker for the detection of CRC, and CRC specific-RNAs combined with proteins in plasma EVs could be unexpected biomarker panels for CRC diagnosis.

### Electronic supplementary material

Below is the link to the electronic supplementary material.

Supplementary Material 1

Supplementary Material 2

Supplementary Material 3

Supplementary Material 4

Supplementary Material 5

Supplementary Material 6

Supplementary Material 7

Supplementary Material 8

Supplementary Material 9

Supplementary Material 10

## Data Availability

All data generated or analyzed during this study are included in this published article and its supplementary information files.

## Abbreviations

CRC: Colorectal cancer
USPSTF: US preventive services task force
CEA: Carcinoembryonic antigen
AUC: Area under the curve
ROC: Receiver operator characteristic
QUADAS-2: Quality Assessment of Diagnostic Accuracy Studies 2
miRNA: MicroRNA
LncRNA: Long non-coding RNAs
cirRNAs: Circular RNAs
mRNAs: Message RNA
UC: Ultralcentrifugation
