# Supplementary figures and images for: Extracellular vesicle biomarkers in circulation for colorectal cancer detection: a systematic review and meta-analysis
Source: BMC Cancer. 2024 May 22;24:623. doi: 10.1186/s12885-024-12312-8 (PMC11110411; doi:10.1186/s12885-024-12312-8)

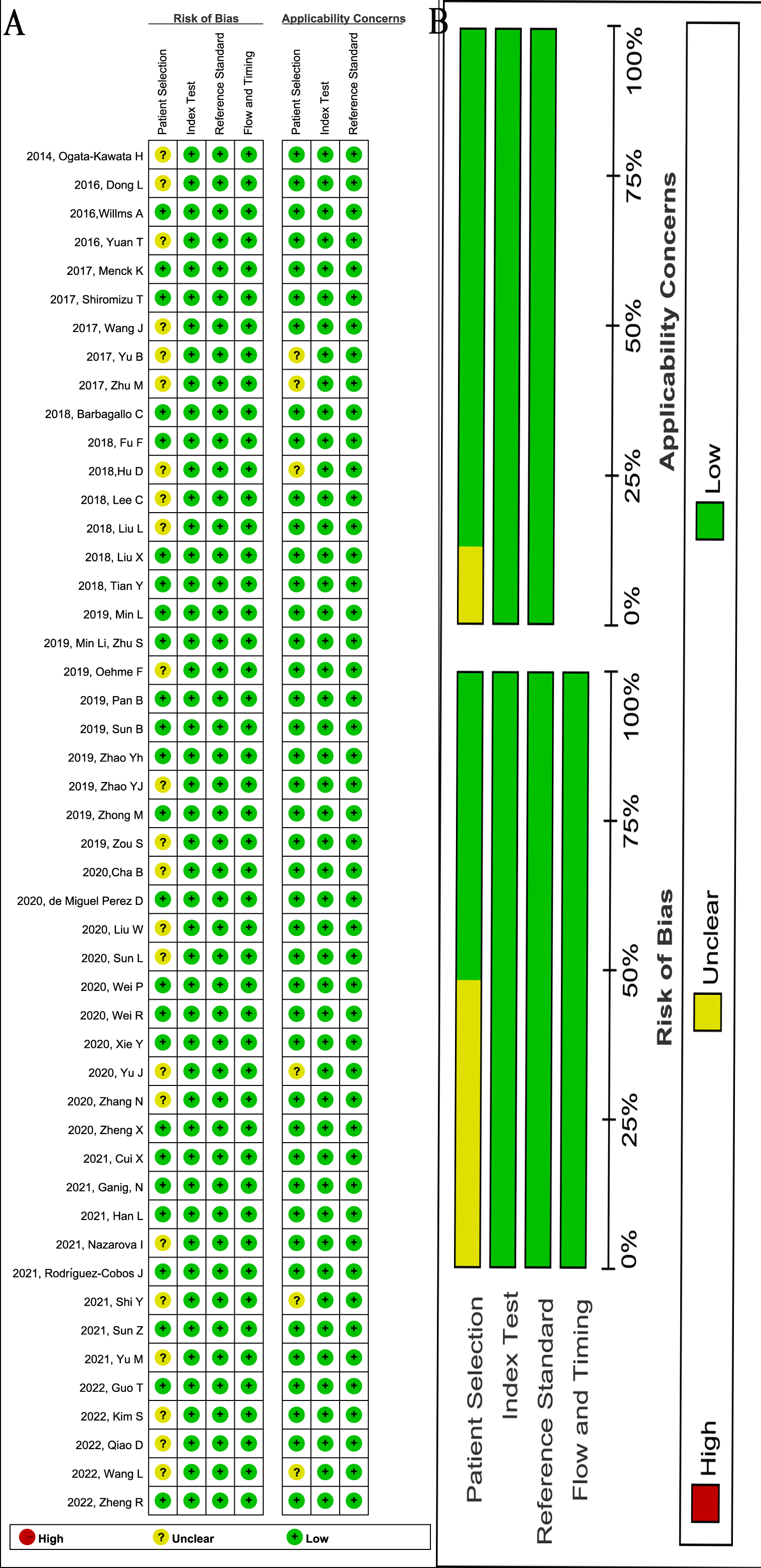

Supplement: Supplementary file 1 — Supplementary Material 1 [file 12885_2024_12312_MOESM1_ESM.tif]

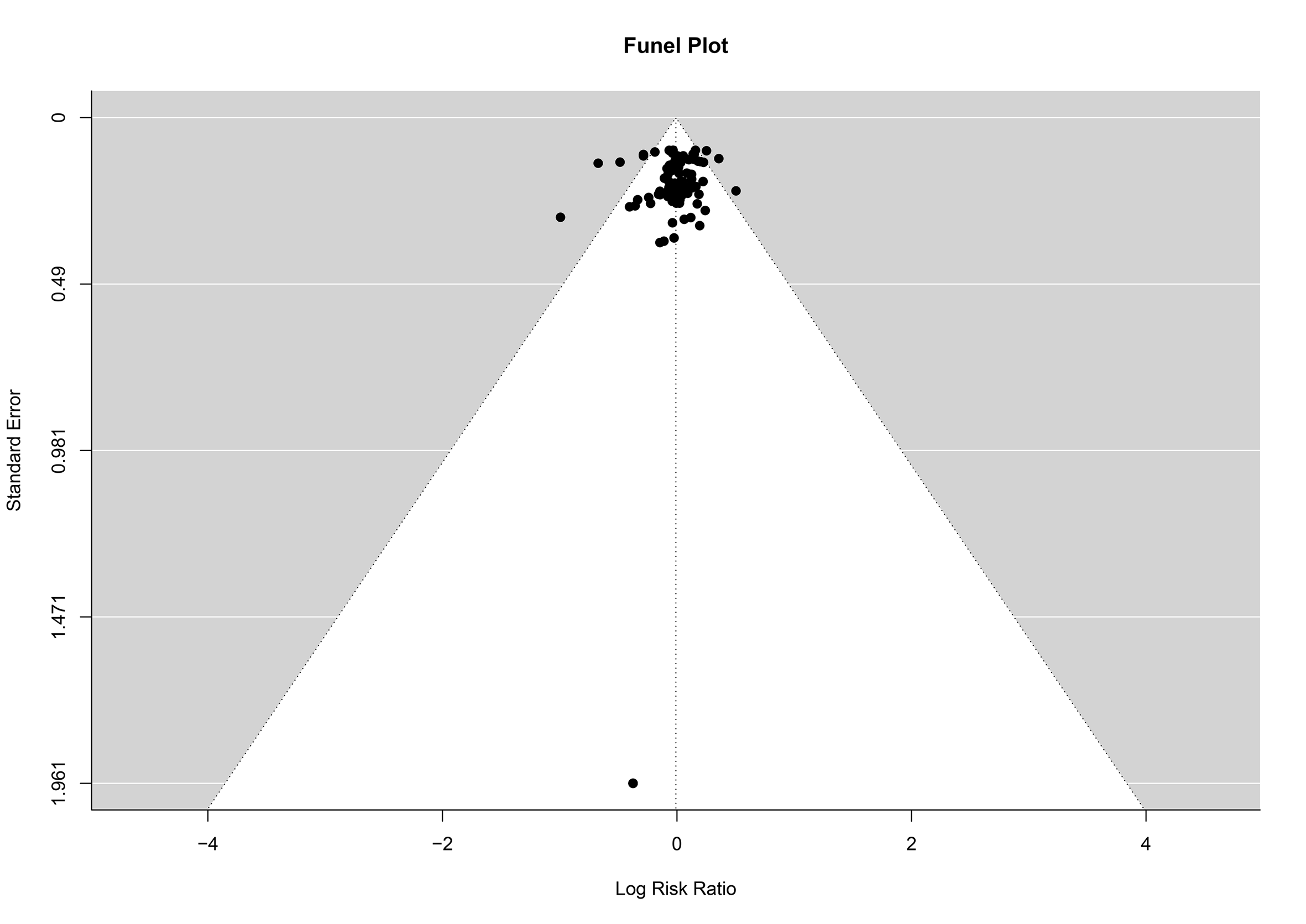

Supplement: Supplementary file 2 — Supplementary Material 2 [file 12885_2024_12312_MOESM2_ESM.tif]

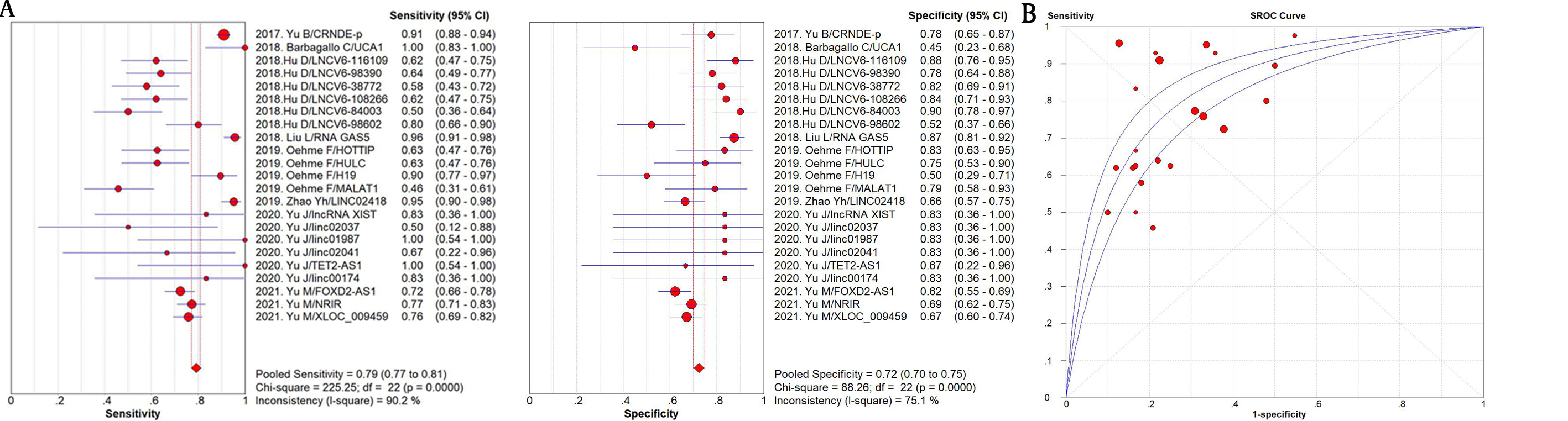

Supplement: Supplementary file 3 — Supplementary Material 3 [file 12885_2024_12312_MOESM3_ESM.tif]

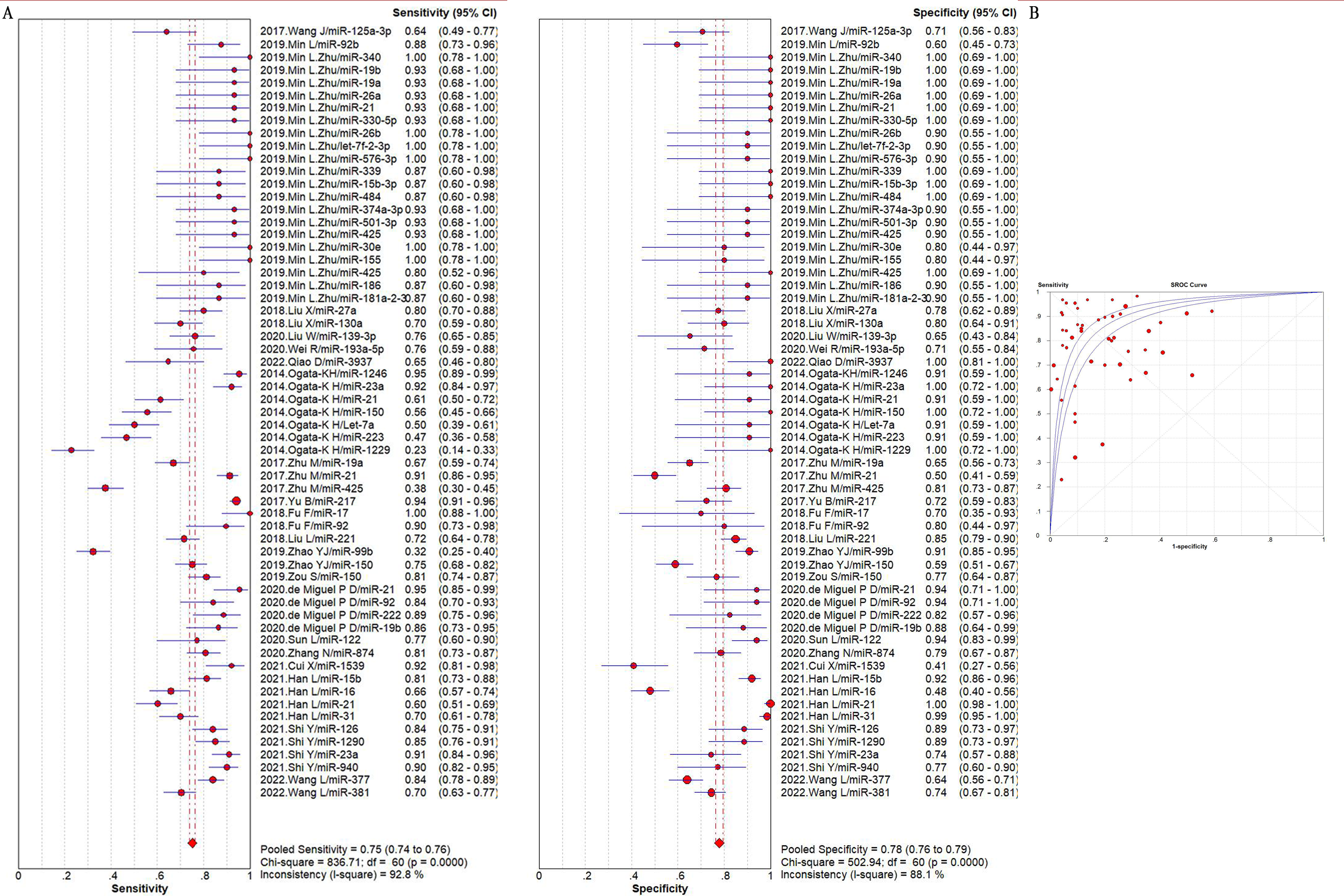

Supplement: Supplementary file 4 — Supplementary Material 4 [file 12885_2024_12312_MOESM4_ESM.tif]
