# Supplementary material for: Extracellular vesicle biomarkers in circulation for colorectal cancer detection: a systematic review and meta-analysis
Source: BMC Cancer. 2024 May 22;24:623. doi: 10.1186/s12885-024-12312-8 (PMC11110411; doi:10.1186/s12885-024-12312-8)
Supplement: Supplementary file 5 — Supplementary Material 5 [file 12885_2024_12312_MOESM5_ESM.docx]

| **Supplementary table 1** Diagnostic performance of RNAs in extracellular vesicles for colorectal cancer | | | | | | | | | | | | |
| --- | --- | --- | --- | --- | --- | --- | --- | --- | --- | --- | --- | --- |
| **Study** | **Country** | **Cases vs Controls** | | | **Specimen** | **Stage** | **Status Controls** | **Detection Method** | **markers** | **SEN%** | **SPE%** | **AUC** |
|  |  | **Number** | **Age** | **Male (%)** |  |  |  |  |  |  |  |  |
|  |  |  |  |  |  |  |  |  | **miRNAs** |  |  |  |
| 2014, Ogata-Kawata H^b^ | Japan | 88/11 | /51 | 73/63 | serum | Ⅰ-Ⅳ | HC | qPCR | miR-1246 | 96 | 91 | 0.948 |
|  |  |  |  |  |  |  |  |  | miR-23a | 92 | 100 | 0.953 |
|  |  |  |  |  |  |  |  |  | miR-21 | 61 | 91 | 0.798 |
|  |  |  |  |  |  |  |  |  | miR-150 | 56 | 100 | 0.758 |
|  |  |  |  |  |  |  |  |  | Let-7a | 50 | 91 | 0.670 |
|  |  |  |  |  |  |  |  |  | miR-223 | 47 | 91 | 0.716 |
|  |  |  |  |  |  |  |  |  | miR-1229 | 23 | 100 | 0.614 |
|  |  | 20/11 | NA | 55/63 |  | Ⅰ |  |  | miR-1246 | 90 | / | / |
|  |  |  |  |  |  |  |  |  | miR-23a | 95 | / | / |
| 2016, Yuan T^a^ | USA | 25/50 | /54 | /50 | plasma | Ⅰ | HC | Small RNA Chip | miR-1343-3p | 60 | 58 | 0.590 |
|  |  |  |  |  |  |  |  |  | miR-125a | 60 | 64 | 0.620 |
|  |  | 25/50 | /54 | /50 |  | Ⅱ |  |  | miR-1343-3p | 68 | 76 | 0.720 |
|  |  |  |  |  |  |  |  |  | miR-125a | 76 | 76 | 0.760 |
|  |  | 25/50 | /54 | /50 |  | Ⅲ |  |  | miR-1343-3p | 64 | 82 | 0.730 |
|  |  |  |  |  |  |  |  |  | miR-125a | 64 | 84 | 0.740 |
|  |  | 25/50 | /54 | /50 |  | Ⅳ |  |  | miR-1343-3p | 80 | 84 | 0.820 |
|  |  |  |  |  |  |  |  |  | miR-125a | 72 | 82 | 0.770 |
|  |  | 100/50 | 55/54 | 50/50 |  | Ⅰ-Ⅳ |  |  | miR-1343-3p | 69 | 74 | 0.720 |
|  |  |  |  |  |  |  |  |  | miR-125a | 69 | 80 | 0.750 |
| 2017, Wang J^b^ | China | 50/50 | 62/ | 64/ | plasma | Ⅰ-Ⅱb | HC | qPCR | miR-125a-3p | 64^*^ | 71^*^ | 0.685 |
| 2017, Yu B^b^ | China | 410/58 | NA | 62/ | serum | Ⅰ-Ⅳ | AD | qPCR | miR-217 | 94^*^ | 73^*^ | 0.864 |
|  |  |  |  |  |  |  |  |  |  |  |  |  |
|  |  |  | | |  |  |  |  |  |  |  |  |
| **Table 1 continued** | | | | | | | | | | | | |
| **Study** | **Country** | **Cases vs Controls** | | | **Specimen** | **Stage** | **Status Controls** | **Detection Method** | **markers** | **SEN%** | **SPE%** | **AUC** |
|  |  | **Number** | **Age** | **Male (%)** |  |  |  |  |  |  |  |  |
| 2017, Zhu M^b^ | China | 166/120 | NA | 60/53 | serum | Ⅰ-Ⅲ | HC | qPCR | miR-19a | 67^*^ | 65^*^ | 0.685 |
|  |  |  |  |  |  |  |  |  | miR-21 | 91^*^ | 50^*^ | 0.773 |
|  |  |  |  |  |  |  |  |  | miR-425 | 38^*^ | 81^*^ | 0.614 |
| 2018, Fu F^b^ | China | 29/10 | NA | 55/50 | serum | Ⅰ-Ⅳ | HC | qPCR | miR-17 | 100^*^ | 73^*^ | 0.897 |
|  |  |  |  |  |  |  |  |  | miR-92 | 90^*^ | 79^*^ | 0.845 |
| 2018, Liu X^b^ | China | 40/40 |  |  |  | Ⅰ | HC | qPCR | miR-27 | 75 | 78 | 0.773 |
|  |  |  |  |  |  |  |  |  | miR-130a | 83 | 63 | 0.742 |
|  |  | 80/40 | 51/53 | 66/63 |  | Ⅰ-Ⅳ | HC |  | miR-27a | 80 | 78 | **0.820** |
|  |  |  |  |  |  |  |  |  | miR-130a | 70 | 80 | **0.787** |
|  |  | 50/100 | 52/ | 63/ |  | Ⅰ | HC+AD |  | miR-27 | 80 | 78 | **0.746** |
|  |  |  |  |  |  |  |  |  | miR-130a | 70 | 80 | **0.697** |
| 2018, Liu L^b^ | China | 158/173 | 57/ | 60/ | serum | Ⅰ-Ⅳ | HC | qPCR | miR-221 | 71^*^ | 85^*^ | 0.827 |
| 2019, Zhao YJ^b^ | China | 165/153 | NA | 67/ | serum | Ⅰ-Ⅳ | HC | qPCR | miR-99b | 32 | 91 | 0.628 |
|  |  |  |  |  |  |  |  |  | miR-150 | 75 | 59 | 0.707 |
|  |  | 72/153 | NA | / |  | Ⅰ-Ⅱ | HC | qPCR | miR-99b | 33 | 92 | 0.652 |
|  |  |  |  |  |  |  |  |  | miR-150 | 81 | 60 | 0.736 |
| 2019, Min L^b^ | China | 40/52 | NA | 75/69 | plasma | Ⅰ-Ⅳ | HC | qPCR | miR-92b | 88^*^ | 60^*^ | 0.793 |
|  |  | 12/52 | NA | /69 |  | Ⅱ-Ⅲ |  |  |  | 88^*^ | 68^*^ | 0.830 |
|  |  | 40/74 | NA | 75/80 |  | Ⅰ-Ⅳ | HC+AD |  |  | 75^*^ | 61^*^ | 0.734 |
| 2019, Zou S^b^ | China | 133/60 | NA | 37/ | serum | Ⅰ-Ⅳ | HC | qPCR | miR-150 | 81 | 76 | 0.870 |
|  |  |  |  |  |  |  |  |  |  |  |  |  |
|  |  |  |  |  |  |  |  |  |  |  |  |  |
| **Table 1 continued** | | | | | | | | | | | | |
| **Study** | **Country** | **Cases vs Controls** | | | **Specimen** | **Stage** | **Status Controls** | **Detection Method** | **markers** | **SEN%** | **SPE%** | **AUC** |
|  |  | **Number** | **Age** | **Male (%)** |  |  |  |  |  |  |  |  |
| 2019, Min L, Zhu S^b^ | China | 58/76 | NA | / | plasma | 0 | GIS | qPCR | let-7b-3p | / | / | **0.792** |
|  |  |  |  |  |  |  |  |  | miR-150-3p | / | / | **0.686** |
|  |  |  |  |  |  |  |  |  | miR-145-3p | / | / | **0.692** |
|  |  |  |  |  |  |  |  |  | miR-139-3p | / | / | **0.679** |
|  |  | 15/10 | NA | / |  |  |  |  | miR-340 | 100 | 100 | / |
|  |  |  |  |  |  |  |  |  | miR-19b | 93 | 100 | / |
|  |  |  |  |  |  |  |  |  | miR-19a | 93 | 100 | / |
|  |  |  |  |  |  |  |  |  | miR-26a | 93 | 100 | / |
|  |  |  |  |  |  |  |  |  | miR-21 | 93 | 100 | / |
|  |  |  |  |  |  |  |  |  | miR-330-5p | 93 | 100 | / |
|  |  |  |  |  |  |  |  |  | miR-26b | 100 | 90 | / |
|  |  |  |  |  |  |  |  |  | let-7f-2-3p | 100 | 90 | / |
|  |  |  |  |  |  |  |  |  | miR-576-3p | 100 | 90 | / |
|  |  |  |  |  |  |  |  |  | miR-339 | 87 | 100 | / |
|  |  |  |  |  |  |  |  |  | miR-15b-3p | 87 | 100 | / |
|  |  |  |  |  |  |  |  |  | miR-484 | 87 | 100 | / |
|  |  |  |  |  |  |  |  |  | miR-374a-3p | 93 | 90 | / |
|  |  |  |  |  |  |  |  |  | miR-501-3p | 93 | 90 | / |
|  |  |  |  |  |  |  |  |  | miR-425 | 93 | 90 | / |
|  |  |  |  |  |  |  |  |  | miR-30e | 100 | 80 | / |
|  |  |  |  |  |  |  |  |  |  |  |  |  |
|  |  |  |  |  |  |  |  |  |  |  |  |  |
|  |  |  |  |  |  |  |  |  |  |  |  |  |
| **Table 1 continued** | | | | | | | | | | | | |
| **Study** | **Country** | **Cases vs Controls** | | | **Specimen** | **Stage** | **Status Controls** | **Detection Method** | **markers** | **SEN%** | **SPE%** | **AUC** |
|  |  | **Number** | **Age** | **Male (%)** |  |  |  |  |  |  |  |  |
| 2019, Min L, Zhu S^b^ | China | 15/10 | NA | / | plasma | 0 | NC | qPCR | miR-155 | 100 | 80 | / |
|  |  |  |  |  |  |  |  |  | miR-425 | 80 | 100 | / |
|  |  |  |  |  |  |  |  |  | miR-186 | 87 | 90 | / |
|  |  |  |  |  |  |  |  |  | miR-181a-2-3p | 87 | 90 | / |
| 2020, de Miguel Perez D^a^ | Spain | 44/17 | NA | 68/59 | serum | Ⅳ | HC | qPCR | miR-21 | 95^*^ | 94^*^ | 0.981 |
|  |  |  |  |  |  |  |  |  | miR-92 | 85^*^ | 95^*^ | 0.951 |
|  |  |  |  |  |  |  |  |  | miR-222 | 88^*^ | 82^*^ | 0.896 |
|  |  |  |  |  |  |  |  |  | miR-19b | 86^*^ | 88^*^ | 0.886 |
| 2020, Liu W^b^ | China | 80/23 | 66/ | 58/ | plasma | Ⅰ-Ⅳ | HC | qPCR | miR-139-3p | 76^*^ | 65^*^ | 0.726 |
|  |  | 38/23 | NA | / |  | Ⅳ |  |  |  | 68^*^ | 76^*^ | 0.766 |
| 2020, Sun L^b^ | China | 35/50 | NA | / | serum | Ⅳ | HC | qPCR | miR-122 | 77^*^ | 94^*^ | **0.890** |
| 2020, Wei R^b^ | China | 37/42 | NA | 76/76 | plasma | Ⅰ-Ⅲ | HC | qPCR | miR-193a-5p | 75^*^ | 72^*^ | 0.759 |
|  |  | 37/22 | NA | 76/86 |  |  | AD |  |  | 68^*^ | 69^*^ | 0.740 |
|  |  | 37/64 | NA | / |  |  | HC+AD |  |  | 74^*^ | 68^*^ | 0.752 |
|  |  | 9/42 | NA | /76 |  | Ⅱ-Ⅲ | HC |  |  | 76^*^ | 84^*^ | 0.823 |
| 2020, Zhang N^b^ | China | 125/70 | NA | 61/ | serum | Ⅰ-Ⅳ | HC | qPCR | miR-874 | 81 | 79 | 0.818 |
|  |  | 125/45 | NA | 61/ |  |  | AD |  |  | 77 | 63 | 0.729 |
| 2021, Cui X^b^ | China | 51/49 | 59/56 | 57/59 | serum | Ⅰ-Ⅳ | HC | qPCR | miR-1539 | 92 | 41 | 0.673 |
| 2021, Han L^b^ | China | 123/150 | 52/52 | 48/51 | serum | / | HC | qPCR | miR-15b | 81 | 92 | 0.860 |
|  |  |  |  |  |  |  |  |  | miR-16 | 66^*^ | 48^*^ | 0.580 |
|  |  |  |  |  |  |  |  |  | miR-21 | 60^*^ | 100^*^ | 0.750 |
|  |  |  |  |  |  |  |  |  | miR-31 | 70^*^ | 99^*^ | 0.750 |
|  |  |  |  |  |  |  |  |  |  |  |  |  |
| **Table 1 continued** | | | | | | | | | | | | |
| **Study** | **Country** | **Cases vs Controls** | | | **Specimen** | **Stage** | **Status Controls** | **Detection Method** | **markers** | **SEN%** | **SPE%** | **AUC** |
|  |  | **Number** | **Age** | **Male (%)** |  |  |  |  |  |  |  |  |
| 2021, Han L^b^ | China | 123/117 | 52/52 | 48/50 |  |  | AD |  | miR-15b | 80^*^ | 86^*^ | 0.790 |
|  |  |  |  |  |  |  |  |  | miR-16 | 79 | 72 | 0.840 |
|  |  |  |  |  |  |  |  |  | miR-21 | 60^*^ | 100^*^ | 0.660 |
|  |  |  |  |  |  |  |  |  | miR-31 | 74^*^ | 51^*^ | 0.550 |
| 2021, Shi Y^b^ | China | 88/11 | /51 | 73/63 | serum | Ⅰ-Ⅳ | HC | qPCR | miR-654 | / | / | 0.980 |
|  |  |  |  |  |  |  |  |  | miR-181d | / | / | 0.830 |
|  |  |  |  |  |  |  |  |  | miR-548c | / | / | 0.870 |
|  |  | 30/35 | NA | /63 | serum | Ⅰ | HC | qPCR | miR-126 | **87** | **77** | **0.900** |
|  |  |  |  |  |  |  |  |  | miR-1290 | **83** | **86** | **0.890** |
|  |  |  |  |  |  |  |  |  | miR-23a | **90** | **74** | **0.850** |
|  |  |  |  |  |  |  |  |  | miR-940 | **90** | **71** | **0.830** |
|  |  | 100/35 | NA | 63/63 | serum | Ⅰ-Ⅳ |  |  | miR-126 | **84** | **89** | **0.940** |
|  |  |  |  |  |  |  |  |  | miR-1290 | **85** | **89** | **0.920** |
|  |  |  |  |  |  |  |  |  | miR-23a | **91** | **74** | **0.890** |
|  |  |  |  |  |  |  |  |  | miR-940 | **90** | **77** | **0.880** |
| 2022, Qiao D^b^ | China | 34/18 | 64/ | 62/ | plasma | Ⅰ-Ⅳ | HC | qPCR | miR-3937 | 65^*^ | 100^*^ | 0.827 |
| 2022, Wang L^b^ | China | 175/172 | NA | 65/ |  | Ⅰ-Ⅳ | HC | qPCR | miR-377 | 84 | 64 | 0.798 |
|  |  |  |  |  |  |  |  |  | miR-381 | 70 | 74 | 0.792 |
|  |  | 69/172 | NA | 61/ |  | Ⅰ-Ⅱb |  |  | miR-377 | 83 | 64 | 0.798 |
|  |  |  |  |  |  |  |  |  | miR-381 | 73 | 74 | 0.803 |
| 2022, Kim S^b^ | Korea | 21/5 | NA | / | plasma | / | HC | NALFA | miR-92a | 81^*^ | 99^*^ | 0.895 |
|  |  |  |  |  |  |  |  |  | miR-141 | 74^*^ | 100^*^ | 0.781 |
|  |  |  |  |  |  |  |  |  |  |  |  |  |
| **Table 1 continued** | | | | | | | | | | | | |
| **Study** | **Country** | **Cases vs Controls** | | | **Specimen** | **Stage** | **Status Controls** | **Detection Method** | **markers** | **SEN%** | **SPE%** | **AUC** |
|  |  | **Number** | **Age** | **Male (%)** |  |  |  |  |  |  |  |  |
|  |  |  |  |  |  |  |  |  | **LcnRNA** |  |  |  |
| 2017, Yu B^b^ | China | 410/58 | NA | 62/ | serum | Ⅰ-Ⅳ | AD | qPCR | CRNDE-p | 91^*^ | 77^*^ | 0.854 |
| 2018, Barbagallo C^b^ | Italy | 20/20 | NA | / | serum | / | HC | qPCR | UCA1 | 100 | 43 | 0.719 |
| 2018,Hu D^b^ | China | 50/50 | NA | / | plasma | Ⅰ-Ⅳ | HC | qPCR | LNCV6-116109 | 62^*^ | 88^*^ | 0.805 |
|  |  |  |  |  |  |  |  |  | LNCV6-98390 | 65^*^ | 77^*^ | 0.709 |
|  |  |  |  |  |  |  |  |  | LNCV6-38772 | 57^*^ | 83^*^ | 0.746 |
|  |  |  |  |  |  |  |  |  | LNCV6-108266 | 62^*^ | 84^*^ | 0.729 |
|  |  |  |  |  |  |  |  |  | LNCV6-84003 | 51^*^ | 90^*^ | 0.736 |
|  |  |  |  |  |  |  |  |  | LNCV6-98602 | 81^*^ | 51^*^ | 0.680 |
| 2018, Liu L^b^ | China | 158/173 | 57/ | 60/ | serum | Ⅰ-Ⅳ | HC | qPCR | RNA GAS5 | 96^*^ | 88^*^ | 0.964 |
| 2019, Oehme F^b^ | Germany | 48/24 | NA | / | plasma | / | HC | qPCR | HOTTIP | 63^*^ | 83^*^ | **0.750** |
|  |  |  |  |  |  |  |  |  | HULC | 62^*^ | 75^*^ | **0.710** |
|  |  |  |  |  |  |  |  |  | H19 | 90^*^ | 49^*^ | **0.720** |
|  |  |  |  |  |  |  |  |  | MALAT1 | 46^*^ | 79^*^ | **0.620** |
| 2019, Zhao Yh^b^ | China | 125/125 | NA | 55/ | serum | 0-Ⅳ | HC | qPCR | LINC02418 | 95 | 66 | 0.898 |
| 2020, Yu J^b^ | China | 6/6 | NA | / | serum | / | HC | qPCR | lncRNA XIST | 88 | 90 | 0.864 |
|  |  |  |  |  |  |  |  |  | lnc02037 | 56 | 83 | 0.698 |
|  |  |  |  |  |  |  |  |  | lnc01987 | 93 | 78 | 0.856 |
|  |  |  |  |  |  |  |  |  | linc02041 | 62 | 90 | 0.774 |
|  |  |  |  |  |  |  |  |  | TET2-AS1 | 96 | 71 | 0.828 |
|  |  |  |  |  |  |  |  |  | linc00174 | 85 | 78 | 0.849 |
|  |  |  |  |  |  |  |  |  |  |  |  |  |
|  |  |  |  |  |  |  |  |  |  |  |  |  |
| **Table 1 continued** | | | | | | | | | | | | |
| **Study** | **Country** | **Cases vs Controls** | | | **Specimen** | **Stage** | **Status Controls** | **Detection Method** | **markers** | **SEN%** | **SPE%** | **AUC** |
|  |  | **Number** | **Age** | **Male (%)** |  |  |  |  |  |  |  |  |
| 2021, Yu M^b^ | China | 203/201 | NA | 67/ | serum | Ⅰ-Ⅳ | HC | qPCR | FOXD2-AS1 | 73 | 62 | 0.728 |
|  |  |  |  |  |  |  |  |  | NRIR | 77 | 69 | 0.660 |
|  |  |  |  |  |  |  |  |  | XLOC_009459 | 76 | 67 | 0.682 |
|  |  | 80/201 | NA | / |  | Ⅰ-Ⅱ |  |  | FOXD2-AS1 | 71 | 59 | 0.743 |
|  |  |  |  |  |  |  |  |  | NRIR | 69 | 70 | 0.660 |
|  |  |  |  |  |  |  |  |  | XLOC_009459 | 73 | 66 | 0.689 |
|  |  |  |  |  |  |  |  |  | **mRNA** |  |  |  |
| 2020,Cha B^a^ | Korea | 10/5 | 69/ | 50/ | serum | Ⅱ-Ⅳ | HC | qPCR | VEGF | 80 | 80 | 0.780 |
|  |  |  |  |  |  |  |  |  | CD133 | 80 | 100 | 0.860 |
|  |  |  |  |  |  |  |  |  | CD24 | 50 | 80 | 0.630 |
|  |  |  |  |  |  |  |  |  | MYC | 40 | 100 | 0.560 |
|  |  |  |  |  |  |  |  |  | CK19 | 40 | 100 | 0.540 |
| 2021, Rodríguez-Cobos J^a^ | Spain | 42/29 | 71/59 | 52/34 | plasma | Ⅰ-Ⅳ | HC | qPCR | ∆Np73 | 62 | 79 | 0.679 |
|  |  |  |  |  |  |  |  |  | ∆133p53 | 48 | 81 | 0.641 |
|  |  | 42/49 | 71/62 | 52/49 |  |  | AD |  | ∆Np73 | 70 | 72 | 0.709 |
|  |  |  |  |  |  |  |  |  | ∆133p53 | 48 | 83 | 0.644 |
|  |  |  |  |  |  |  |  |  | **circRNA** |  |  |  |
| 2018, Barbagallo C^b^ | Italy | 20/20 | NA | / | serum | / | HC | qPCR | circHIPK3 | 71 | 80 | 0.770 |
| 2019, Pan B^b^ | China | 70/35 | /60 | /54 | serum | Ⅰ-Ⅱb | HC | qPCR | circ-0004771 | **81** | **80** | **0.860** |
|  |  |  |  |  |  |  | BID |  |  | **81** | **74** | **0.810** |
|  |  | 110/35 | 60/60 | 69/54 |  | Ⅰ-Ⅳ | HC |  |  | **81** | **83** | **0.880** |
|  |  |  |  |  |  |  |  |  |  |  |  |  |
|  |  |  |  |  |  |  |  |  |  |  |  |  |
| **Table 1 continued** | | | | | | | | | | | | |
| **Study** | **Country** | **Cases vs Controls** | | | **Specimen** | **Stage** | **Status Controls** | **Detection Method** | **markers** | **SEN%** | **SPE%** | **AUC** |
|  |  | **Number** | **Age** | **Male (%)** |  |  |  |  |  |  |  |  |
| 2020, Xie Y^b^ | China | 58/58 | 65/55 | 67/48 | serum | Ⅰ-Ⅳ | HC | qPCR | circ-PNN | **90** | **69** | **0.826** |
|  |  | 36/58 |  |  |  | Ⅰ-Ⅱ |  |  |  | **92** | **69** | **0.854** |
| 2022, Zheng R^b^ | China | 112/60 | NA | / | plasma | / | NC | RNA-Seq | circLPAR1 | 68^*^ | 100^*^ | 0.858 |

SENs, SPEs and AUCs in bold fonts represent results from validation set (non-bold fonts represent results without validation)

SEN, sensitivity; SPE, specificity; AUC, area under the curve; HC, healthy control; BID, benign intestinal diseases; NC, noncancerous; AD, adenoma; NALFA, DNA barcode-based nucleic acid lateral flow assay; NA, not available .

^*^ represent estimated sensitivity and specificity

^a^ represent markers extracted from extracellular vesicles;

^b^ represent markers extracted from exosomes;

^c^ represent markers extracted from microparticles;

^d^ represent markers extracted from microvesicles.
