# Supplementary material for: Extracellular vesicle biomarkers in circulation for colorectal cancer detection: a systematic review and meta-analysis
Source: BMC Cancer. 2024 May 22;24:623. doi: 10.1186/s12885-024-12312-8 (PMC11110411; doi:10.1186/s12885-024-12312-8)
Supplement: Supplementary file 6 — Supplementary Material 6 [file 12885_2024_12312_MOESM6_ESM.docx]

| **Supplementary table 2** Diagnostic performance of proteins in extracellular vesicles for colorectal cancer | | | | | | | | | | | | |
| --- | --- | --- | --- | --- | --- | --- | --- | --- | --- | --- | --- | --- |
| **Study** | **Country** | **Cases vs Controls** | | | **Specimen** | **Stage** | **Status Controls** | **Detection Method** | **markers** | **SEN%** | **SPE%** | **AUC** |
|  |  | **Number** | **Age** | **Male (%)** |  |  |  |  |  |  |  |  |
| 2016,Willms A^c^ | Germany | 52/55 | 67/28 | 67/66 | serum | Ⅰ-Ⅳ | HC | FACS | EpCAM | 94 | 53 | 0.847 |
| 2017, Menck K^d^ | Germany | 34/62 | /36 | /58 | plasma | / | HC | Flow cytometry | EMMPRIN | 50 | 89 | 0.700 |
| 2017, Shiromizu T^a^ | Japan | 28/28 | 69/55 | 50/57 | serum | Ⅰ | HC | SRM | ANXA11 | 86^*^ | 100^*^ | **0.960** |
|  |  |  |  |  |  |  |  |  | ANXA3 | 95^*^ | 97^*^ | **0.990** |
|  |  |  |  |  |  |  |  |  | ANXA4 | 85^*^ | 97^*^ | **0.940** |
|  |  |  |  |  |  |  |  |  | TNN | 94^*^ | 55^*^ | **0.780** |
|  |  |  |  |  |  |  |  |  | TFRC | 87^*^ | 88^*^ | **0.910** |
|  |  |  |  |  |  |  |  |  | GLUT-1 | / | / | **0.960** |
|  |  |  |  |  |  |  |  |  | C9 | 85^*^ | 81^*^ | **0.910** |
|  |  |  |  |  |  |  |  |  | CD88 | / | / | **0.940** |
|  |  |  |  |  |  |  |  |  | HSPA5 | 94^*^ | 71^*^ | **0.860** |
|  |  |  |  |  |  |  |  |  | ORM1 | 93^*^ | 67^*^ | **0.880** |
|  |  |  |  |  |  |  |  |  | MMP9 | 83^*^ | 96^*^ | **0.970** |
|  |  |  |  |  |  |  |  |  | ANGPT1 | 64^*^ | 80^*^ | **0.740** |
|  |  |  |  |  |  |  |  |  | CEACAM8 | 87^*^ | 93^*^ | **0.980** |
|  |  |  |  |  |  |  |  |  | MUC5B | 88^*^ | 56^*^ | **0.740** |
|  |  |  |  |  |  |  |  |  | GRB2 | 81^*^ | 78^*^ | **0.850** |
|  |  |  |  |  |  |  |  |  | ANXA5 | / | / | **0.980** |
|  |  |  |  |  |  |  |  |  | OLFM4 | 89^*^ | 88^*^ | **0.950** |
|  |  |  |  |  |  |  |  |  | SLC1A5 | 76^*^ | 96^*^ | **0.870** |
|  |  |  |  |  |  |  |  |  | TPP1 | / | / | **0.840** |
|  |  |  |  |  |  |  |  |  |  |  |  |  |
|  |  |  |  |  |  |  |  |  |  |  |  |  |
| **Table 1 continued** | | | | | | | | | | | | |
| **Study** | **Country** | **Cases vs Controls** | | | **Specimen** | **Stage** | **Status Controls** | **Detection Method** | **markers** | **SEN%** | **SPE%** | **AUC** |
|  |  | **Number** | **Age** | **Male (%)** |  |  |  |  |  |  |  |  |
| 2017, Shiromizu T^a^ | Japan | 28/28 | 69/55 | 50/57 | serum | Ⅰ | HC | SRM | HSPA2 | / | / | **0.870** |
|  |  |  |  |  |  |  |  |  | PSMA5 | 90^*^ | 63^*^ | **0.790** |
|  |  |  |  |  |  |  |  |  | LCN2 | 88^*^ | 94^*^ | **0.950** |
|  |  | 28/28 | 76/55 | 50/57 | serum | Ⅱ |  |  | ANXA11 | 100^*^ | 98^*^ | **0.990** |
|  |  |  |  |  |  |  |  |  | ANXA3 | 91^*^ | 100^*^ | **0.990** |
|  |  |  |  |  |  |  |  |  | ANXA4 | 87^*^ | 100^*^ | **0.980** |
|  |  |  |  |  |  |  |  |  | TNN | 62^*^ | 84^*^ | **0.790** |
|  |  |  |  |  |  |  |  |  | TFRC | 88^*^ | 80^*^ | **0.920** |
|  |  |  |  |  |  |  |  |  | GLUT-1 | / | / | **0.980** |
|  |  |  |  |  |  |  |  |  | C9 | 92^*^ | 98^*^ | **0.980** |
|  |  |  |  |  |  |  |  |  | CD88 | / | / | **0.980** |
|  |  |  |  |  |  |  |  |  | HSPA5 | 94^*^ | 71^*^ | **0.920** |
|  |  |  |  |  |  |  |  |  | ORM1 | 74^*^ | 89^*^ | **0.890** |
|  |  |  |  |  |  |  |  |  | MMP9 | 94^*^ | 95^*^ | **0.990** |
|  |  |  |  |  |  |  |  |  | ANGPT1 | 60^*^ | 81^*^ | **0.750** |
|  |  |  |  |  |  |  |  |  | CEACAM8 | 96^*^ | 91 | **0.990** |
|  |  |  |  |  |  |  |  |  | MUC5B | 89 | 76^*^ | **0.880** |
|  |  |  |  |  |  |  |  |  | GRB2 | 88^*^ | 90^*^ | **0.960** |
|  |  |  |  |  |  |  |  |  | ANXA5 | / | / | **0.990** |
|  |  |  |  |  |  |  |  |  | OLFM4 | 91^*^ | 99^*^ | **0.980** |
|  |  |  |  |  |  |  |  |  | SLC1A5 | 68^*^ | 88^*^ | **0.840** |
|  |  |  |  |  |  |  |  |  |  |  |  |  |
|  |  |  |  |  |  |  |  |  |  |  |  |  |
| **Table 1 continued** | | | | | | | | | | | | |
| **Study** | **Country** | **Cases vs Controls** | | | **Specimen** | **Stage** | **Status Controls** | **Detection Method** | **markers** | **SEN%** | **SPE%** | **AUC** |
|  |  | **Number** | **Age** | **Male (%)** |  |  |  |  |  |  |  |  |
| 2017, Shiromizu T^a^ | Japan | 28/28 | 76/55 | 50/57 | serum | Ⅱ | HC | SRM | TPP1 | / | / | **0.880** |
|  |  |  |  |  |  |  |  |  | HSPA2 | / | / | **0.890** |
|  |  |  |  |  |  |  |  |  | PSMA5 | 90^*^ | 63^*^ | **0.660** |
|  |  |  |  |  |  |  |  |  | LCN2 | 90^*^ | 79^*^ | **0.950** |
| 2018，Tian Y^a^ | China | 37/32 | 62/26 | 59/53 | plasma | Ⅰ-Ⅳ | HC | HSFC | CD147 | 83^*^ | 94^*^ | 0.932 |
| 2019, Sun B^b^ | China | 92/32 | NA | / | plasma | Ⅰ-Ⅳ | HC | ELISA | CPNE3 | 67 | 84 | 0.791 |
| 2019, Zhong M^a^ | China | 125/25 | NA | / | serum | Ⅲ | HC | ELISA | SPARC | 97^*^ | 84^*^ | 0.950 |
|  |  |  |  |  |  |  |  |  | LRG1 | 87^*^ | 87^*^ | 0.930 |
| 2020, Zheng X^a^ | China | 12/12 | 68/50 | 58/67 | plasma | Ⅰ-Ⅲb | HC | DIA-MS | FGA | **100** | **100** | **1.000** |
| 2021, Ganig N^b^ | Germany | 48/18 | 67/72 | 69/78 | plasma | 0-Ⅳ | VD | ELISA | QSOX1 | 85 | 83 | 0.904 |
|  |  | 24/18 | /72 | /78 |  | 0-Ⅲ |  |  |  | 74 | 94 | 0.887 |
|  |  | 24/18 | /72 | /78 |  | Ⅳ |  |  |  | 88 | 83 | 0.921 |
| 2021, Sun Z^b^ | China | 30/20 | NA | 57/60 | plasma | / | HC | ELISA | FGB | 68 | 86 | 0.871 |
|  |  |  |  |  |  |  |  |  | β2-GP1 | 72 | 86 | 0.834 |

SENs, SPEs and AUCs in bold fonts represent results from validation set (non-bold fonts represent results without validation)

SEN, sensitivity; SPE, specificity; AUC, area under the curve; HC, healthy control; AD, adenoma; NALFA, DNA barcode-based nucleic acid lateral flow assay; FACS, Fluorescence Activating Cell Sorter; ELISA, Enzyme Linked Immunosorbent Assay; SRM, Selected reaction monitoring; DIA-MS, data-independent acquisition-mass spectrometry; HSFC, high-sensitivity flow cytometer; NA, not available.

^*^ represent estimated sensitivity and specificity;

^a^ represent markers extracted from extracellular vesicles;

^b^ represent markers extracted from exosomes;

^c^ represent markers extracted from microparticles;

^d^ represent markers extracted from microvesicles.
