# Supplementary material for: Extracellular vesicle biomarkers in circulation for colorectal cancer detection: a systematic review and meta-analysis
Source: BMC Cancer. 2024 May 22;24:623. doi: 10.1186/s12885-024-12312-8 (PMC11110411; doi:10.1186/s12885-024-12312-8)
Supplement: Supplementary file 7 — Supplementary Material 7 [file 12885_2024_12312_MOESM7_ESM.docx]

| **Supplementary table 3** Diagnostic performance of biomarker panels in extracellular vesicles for colorectal cancer | | | | | | | | | | | | |
| --- | --- | --- | --- | --- | --- | --- | --- | --- | --- | --- | --- | --- |
| **Study** | **Country** | **Cases vs Controls** | | | **Specimen** | **Stage** | **Status Controls** | **Detection Method** | **markers** | **SEN%** | **SPE%** | **AUC** |
|  |  | **Number** | **Age** | **Male (%)** |  |  |  |  |  |  |  |  |
| 2016, Yuan T^a^ | USA | 25/50 | /54 | /50 | plasma | Ⅰ | HC | Small RNA Chip | panelA | 64 | 72 | 0.680 |
|  |  | 25/50 |  |  |  | Ⅱ |  |  |  | 76 | 78 | 0.770 |
|  |  | 25/50 |  |  |  | Ⅲ |  |  |  | 68 | 88 | 0.780 |
|  |  | 25/50 |  |  |  | Ⅳ |  |  |  | 76 | 86 | 0.810 |
|  |  | 100/50 | 55/54 | 50/50 |  | Ⅰ-Ⅳ |  |  |  | 72 | 80 | 0.760 |
| 2017, Zhu M^b^ | China | 166/120 | NA | 60/53 | serum | Ⅰ-Ⅲ | HC | qPCR | panelB | 84* | 66* | 0.825 |
|  |  | 30/18 | NA | 77/56 |  |  |  |  | panelC | 93* | 61* | **0.830** |
| 2018, Fu F^b^ | China | 29/10 | NA | 55/50 | serum | Ⅰ-Ⅳ | HC | qPCR | panelD | 100* | 75* | 0.910 |
| 2019, Min L, Zhu S^b^ | China | 58/76 | NA | / | plasma | 0 | GIS | qPCR | panelE | 74* | 83* | **0.828** |
|  |  |  |  |  |  |  |  |  | panelF | 81* | 90* | **0.901** |
|  |  |  |  |  |  |  |  |  | panelG | 83* | 89* | **0.927** |
|  |  |  |  |  |  |  |  |  | panelH | 85* | 87* | **0.927** |
| 2018, Liu X^b^ | China | 40/40 |  |  | plasma | Ⅰ | HC | qPCR | panelJ | 83 | 75 | 0.846 |
|  |  | 80/40 | 51 /53 | 66/63 |  | Ⅰ-Ⅳ | HC |  |  | **80** | **90** | **0.898** |
|  |  | 50/100 | 52/ | 63/ |  | Ⅰ | HC+AD |  |  | **80** | **90** | **0.801** |
| 2021, Han L^b^ | China | 81/90 | NA | / | serum | / | HC | qPCR | panelK | **95** | **94** | / |
|  |  | 81/67 | NA | / |  | / | AD |  | panelL | **85** | **82** | / |
| 2021, Shi Y^b^ | China | 30/35 | NA | /63 | serum | Ⅰ | HC | qPCR | panelM | **90** | **83** | **0.920** |
|  |  | 100/35 | NA | 63/63 | serum | Ⅰ-Ⅳ |  |  |  | **90** | **89** | **0.950** |
| 2022, Wang L^b^ | China | 175/172 | NA | 65/ | serum | Ⅰ-Ⅳ | HC | qPCR | panelN | 91 | 59 | 0.807 |
|  |  | 69/172 | NA | 61/ |  | Ⅰ-Ⅱb |  |  |  | 80 | 67 | 0.815 |
| 2022, Kim S^b^ | Korea | 21/5 | NA | / | plasma | / | HC | NALFA | panelO | 95 | 100 | 0.952 |
|  |  |  |  |  |  |  |  |  |  |  |  |  |
| **Table 3 continued** | | | | | | | | | | | | |
| **Study** | **Country** | **Cases vs Controls** | | | **Specimen** | **Stage** | **Status Controls** | **Detection Method** | **markers** | **SEN%** | **SPE%** | **AUC** |
|  |  | **Number** | **Age** | **Male (%)** |  |  |  |  |  |  |  |  |
| 2017, Yu B^b^ | China | 410/58 | NA | 61.5/ | serum | Ⅰ-Ⅳ | AD | qPCR | panelP | 83* | 90* | 0.933 |
| 2018, Barbagallo C^b^ | Italy | 20/20 | NA | / | serum | / | HC | qPCR | panelQ | 93 | 64 | 0.814 |
|  |  |  |  |  |  |  |  |  | panelR | 100 | 70 | 0.900 |
| 2021, Yu M^b^ | China | 203/201 | NA | 67/ | serum | Ⅰ-Ⅳ | HC | qPCR | panelS | 61 | 75 | 0.736 |
|  |  | 80/201 | NA | / |  | Ⅰ-Ⅱ |  |  |  | 67 | 74 | 0.758 |
| 2022, Guo T^a^ | China | 72/80 | 61/60 | 67/68 | plasma | Ⅰ-Ⅳ | HC | exLR-seq Analysis | panelT | 93 | 94 | 0.983 |
|  |  | 53/80 | /60 | /68 |  | Ⅰ-Ⅱ |  |  |  | 95 | 96 | 0.990 |
|  |  | 72/42 | 61/56 | 67/57 |  | Ⅰ-Ⅳ | AD |  |  | 76 | 85 | 0.853 |
|  |  | 53/42 | /60 | /57 |  | Ⅰ-Ⅱ |  |  |  | 86 | 81 | 0.882 |
|  |  | 14/23 | NA | / |  | / |  |  |  | **71** | **96** | **0.947** |
| 2016, Dong L^b^ | China | 30/30 | 61/ | 33/ | serum | Ⅰ-Ⅳ | HC | qPCR | panelU | 69* | 86* | 0.877 |
| 2020,Cha B^a^ | Korea | 10/5 | 69/ | 50/ | serum | Ⅱ-Ⅳ | HC | qPCR | panelV | 100 | 80 | 0.960 |
|  |  |  |  |  |  |  |  |  | panelW | 100 | 80 | 0.960 |
|  |  |  |  |  |  |  |  |  | panelX | 100 | 80 | 0.960 |
|  |  |  |  |  |  |  |  |  | **Protein panels** |  |  |  |
| 2016,Willms A^c^ | Germany | 49/48 | NA | / | serum | Ⅰ-Ⅳ | HC | FACS | panelY | 96 | 55 | 0.860 |
| 2017, Menck K^d^ | Germany | 34/57 | NA | / | plasma | / | HC | Flow cytometry | panelZ | 71 | 86 | 0.830 |
| 2018, Lee C^a^ | Korea | 37/30 | NA | / | plasma | / | HC | ELISA | panela | 76 | 67 | 0.828 |
| 2020, Wei P^a^ | China | 163/46 | 57/43.7 | 63/26 | plasma | 0-Ⅲ | HC | SiMoa EV assays | panelb | 93 | 96 | 0.960 |
|  |  |  |  |  |  |  |  |  | panelc | 100 | 100 | 0.900 |
| 2021, Sun Z^b^ | China | 30/20 | NA | 57/60 | plasma | / | HC | ELISA | paneld | 64 | 94 | 0.915 |
|  |  |  |  |  |  |  |  |  |  |  |  |  |
|  |  |  |  |  |  |  |  |  |  |  |  |  |
| **Table 3 continued** | | | | | | | | | | | | |
| **Study** | **Country** | **Cases vs Controls** | | | **Specimen** | **Stage** | **Status Controls** | **Detection Method** | **markers** | **SEN%** | **SPE%** | **AUC** |
|  |  | **Number** | **Age** | **Male (%)** |  |  |  |  |  |  |  |  |
| 2021,Nazarova I^b^ | Russia | 20/20 | 53/ | 65/ | plasma | Ⅲb-IV | HC | on-bead flow cytometry | panele | 75* | 85* | 0.810 |
|  |  |  |  |  |  |  |  |  | Panelg | / | / | 0.780 |
|  |  |  |  |  |  |  |  |  | Panel1 | / | / | 0.710 |
|  |  |  |  |  |  |  |  |  | Panel2 | / | / | 0.780 |
|  |  |  |  |  |  |  |  |  | Panel3 | / | / | 0.740 |
|  |  |  |  |  |  |  |  |  | Panel4 | / | / | 0.780 |
|  |  |  |  |  |  |  |  |  | panelf | 55* | 94* | 0.790 |
|  |  |  |  |  |  |  |  |  | Panel5 | / | / | 0.780 |
|  |  |  |  |  |  |  |  |  | Panel6 | / | / | 0.790 |
|  |  |  |  |  |  |  |  |  | Panel7 | / | / | 0.750 |
|  |  | 28/30 | 51/ | 54/ |  | Ⅱ-Ⅲb |  |  | panele | 75 | 82 | 0.820 |
| 2017, Shiromizu T^a^ | Japan | 26/26 | 72/59 | 54/65 | serum | Ⅱ | HC | SRM | panelh | 88 | 85 | 0.950 |
|  |  |  |  |  |  |  |  |  | paneli | 87 | 85 | 0.900 |
|  |  |  |  |  |  |  |  |  | panelj | 91 | 83 | 0.950 |
|  |  |  |  |  |  |  |  |  | panelk | 88 | 88 | 0.950 |
|  |  |  |  |  |  |  |  |  | panell | 87 | 85 | 0.910 |
|  |  |  |  |  |  |  |  |  | panelm | 95 | 85 | 0.930 |
|  |  |  |  |  |  |  |  |  | paneln | 80 | 78 | 0.890 |
|  |  |  |  |  |  |  |  |  | panelo | 71 | 73 | 0.800 |
|  |  |  |  |  |  |  |  |  | panelp | 80 | 80 | 0.880 |
|  |  |  |  |  |  |  |  |  |  |  |  |  |
|  |  |  |  |  |  |  |  |  |  |  |  |  |
|  |  |  |  |  |  |  |  |  |  |  |  |  |
| **Table 3 continued** | | | | | | | | | | | | |
| **Study** | **Country** | **Cases vs Controls** | | | **Specimen** | **Stage** | **Status Controls** | **Detection Method** | **markers** | **SEN%** | **SPE%** | **AUC** |
|  |  | **Number** | **Age** | **Male (%)** |  |  |  |  |  |  |  |  |
| 2017, Shiromizu T^a^ | Japan | 26/26 | 72/59 | 54/65 | serum | Ⅱ | HC | SRM | panelq | 82 | 80 | 0.880 |
|  |  |  |  |  |  |  |  |  | panelr | 82 | 79 | 0.850 |
|  |  |  |  |  |  |  |  |  | panels | 83 | 84 | 0.880 |
|  |  |  |  |  |  |  |  |  | panelt | 96 | 92 | 0.970 |

SENs, SPEs and AUCs in bold fonts represent results from validation set (non-bold fonts represent results without validation)

SEN, sensitivity; SPE, specificity; AUC, area under the curve; HC, healthy control; GIS. gastrointestinal symptoms; AD, adenoma; NALFA, DNA barcode-based nucleic acid lateral flow assay; FACS, Fluorescence Activating Cell Sorter; ELISA, Enzyme Linked Immunosorbent Assay; SRM, Selected reaction monitoring.

^*^ represent estimated sensitivity and specificity;

^a^ represent markers extracted from extracellular vesicles;

^b^ represent markers extracted from exosomes;

^c^ represent markers extracted from microparticles;

^d^ represent markers extracted from microvesicles.

panelA, miR-125a/miR-1343-3p/miR-708/miR-381/miR-543/piR_019825; panelB, miR-19a/miR-21/miR-425/miR-122; panelC, miR-19a/miR-21/miR-425; panelD, miR-17/miR-92; panelE, let-7b-3p/miR-139-3p; panelF, let-7b-3p/miR-145-3p; panelG, let-7b-3p/miR-139-3p/miR-145-3p; panelH, let-7b-3p/miR-139-3p/miR-145-3p/miR-150-3p; panelJ, miR-27/miR-130a; panelJ, miR-27/miR-130a; panelK, miR-15b/miR-21/miR-31; panelL, miR-15b/miR-21/miR-16; panelM, miR-126/miR-1290/miR-23a/miR-940; panelN, miR-377/miR-381; panelO, miR-92a/miR-141; panelP, CRNDE-p/miR-217; panelQ, TUG1/UCA1; panelR, circHIPK3/UCA1; panelS, FOXD2-AS1/NRIR/XLOC_009459; panelT, HIST2H2AA4/H2BFS/UQCRHL/XCL2/AC008269.1/DMC1/RAB6D/KLHDC8B/CA3/APOL4/HIST1H2AI/ANKAR/SGMS1/CYP20A1/HIST1H2BB/STK3/CBWD1; panelU, KRTAP5-4/MAGEA3/BCAR4; panelV, VEGF/CD133; panelW, VEGF/CD133/CK19; panelX, VEGF/CD133/CD24; panelY, EpCAM/CD147; panelZ, EMMPRIN/MUC1/EGFR/EpCAM; panela, TSPAN1/CD63; panelb, CD9/CD63; panelc, Epcam/CD63; paneld, FGB/β2-GP1; panele, CD63/FITC; panelg, CD63/CLRN3; panel1, CD63/GCNT3; panel2, CD63/PIGY; panel3, CD63/REG4; panel4, CD63/Mucin12; panelf, CD9/PerCP; panel5, CD9/REG4; panel6, CD9/MEP1A; panel7, CD9/Mucin12; panele, CD63/FITC; panelh, GLUT-1/TFRC; paneli, GLUT-1/ANGPT1; panelj, MMP9/TFRC; panelk, TFRC/LCN2; panel, TFRC/ANGPT1; panelm, TFRC/GRB2; paneln, TFRC/OLFM4; panelo, HSPA5/TPP1; panelp, HSPA5/ANGPT1; panelq, HSPA5/OLFM4; panelr, OLFM4/ANGPT1; panels, OLFM4/TPP1; panelt, TFRC/LCN2/ANGPT1.
