# Supplementary material for: Extracellular vesicle biomarkers in circulation for colorectal cancer detection: a systematic review and meta-analysis
Source: BMC Cancer. 2024 May 22;24:623. doi: 10.1186/s12885-024-12312-8 (PMC11110411; doi:10.1186/s12885-024-12312-8)
Supplement: Supplementary file 9 — Supplementary Material 9 [file 12885_2024_12312_MOESM9_ESM.docx]

| **supplementary table 5** Summary of studies reporting significant associations of RNAs in colorectal cancer | | | | | | | | | | | | | | | | | | | | | | | | | | | | | | | | | | | | | | |
| --- | --- | --- | --- | --- | --- | --- | --- | --- | --- | --- | --- | --- | --- | --- | --- | --- | --- | --- | --- | --- | --- | --- | --- | --- | --- | --- | --- | --- | --- | --- | --- | --- | --- | --- | --- | --- | --- | --- |
| **RNAs** | **(61)** | **(60)** | **(58)** | **(55)** | **(54)** | **(53)** | **(52)** | **(51)** | **(48)** | **(47)** | **(50)** | **(45)** | **(44)** | **(43)** | **(42)** | **(40)** | **(39)** | **(37)** | **(35)** | **(34)** | **(33)** | **(31)** | **(30)** | **(29)** | **(28)** | **(36)** | **(26)** | **(24)** | **(22)** | **(21)** | **(19)** | **(18)** | **(17)** | **(16)** | **(15)** | **(14)** | **Number of**  **studies** | |
| miR-21 | ↑○ |  |  |  |  | ↑△ |  |  |  |  |  |  | ↓○ |  |  |  |  |  | ↑○ |  |  |  |  |  |  |  |  | ↑△ |  |  |  |  |  |  |  |  | 5 | |
| miR-150 | ↑○ |  |  |  |  |  |  |  |  |  |  |  |  |  |  |  | ↓○ | ↓○ |  |  |  |  |  |  |  |  |  |  |  |  |  |  |  |  |  |  | 3 | |
| miR-122 |  |  |  |  |  | △ |  |  |  |  |  |  |  |  |  |  |  |  |  |  | ↑○ |  |  |  |  |  |  |  |  |  |  |  |  |  |  |  | 2 | |
| miR-139-3p |  |  |  |  |  |  |  |  |  |  |  |  | ↑△ |  |  |  |  |  |  | ↓○ |  |  |  |  |  |  |  |  |  |  |  |  |  |  |  |  | 2 | |
| miR-19a |  |  |  |  |  | ↑△ |  |  |  |  |  |  | ↓○ |  |  |  |  |  |  |  |  |  |  |  |  |  |  |  |  |  |  |  |  |  |  |  | 2 | |
| miR-19b |  |  |  |  |  |  |  |  |  |  |  |  | ↓○ |  |  |  |  |  | ↑○ |  |  |  |  |  |  |  |  |  |  |  |  |  |  |  |  |  | 2 | |
| miR-23a | ↑○ |  |  |  |  |  |  |  |  |  |  |  |  |  |  |  |  |  |  |  |  |  |  |  |  |  |  |  |  | ↑△ |  |  |  |  |  |  | 2 | |
| miR-381 |  |  | △ |  |  |  |  |  |  |  |  |  |  |  |  |  |  |  |  |  |  |  |  |  |  |  |  |  |  |  |  |  |  |  | ↓△ |  | 2 | |
| miR-425 |  |  |  |  |  | ↑△ |  |  |  |  |  |  | ↓○ |  |  |  |  |  |  |  |  |  |  |  |  |  |  |  |  |  |  |  |  |  |  |  | 2 | |
| miR-92 |  |  |  |  |  |  |  | ↑△ |  |  |  |  |  |  |  |  |  |  | ↑○ |  |  |  |  |  |  |  |  |  |  |  |  |  |  |  |  |  | 2 | |
| miR-1229 | ↑○ |  |  |  |  |  |  |  |  |  |  |  |  |  |  |  |  |  |  |  |  |  |  |  |  |  |  |  |  |  |  |  |  |  |  |  | 1 | |
| miR-1246 | ↑○ |  |  |  |  |  |  |  |  |  |  |  |  |  |  |  |  |  |  |  |  |  |  |  |  |  |  |  |  |  |  |  |  |  |  |  | 1 | |
| miR-125a |  |  | ↑△ |  |  |  |  |  |  |  |  |  |  |  |  |  |  |  |  |  |  |  |  |  |  |  |  |  |  |  |  |  |  |  |  |  | 1 | |
| miR-125a-3p |  |  |  | ↑○ |  |  |  |  |  |  |  |  |  |  |  |  |  |  |  |  |  |  |  |  |  |  |  |  |  |  |  |  |  |  |  |  | 1 | |
| miR-126 |  |  |  |  |  |  |  |  |  |  |  |  |  |  |  |  |  |  |  |  |  |  |  |  |  |  |  |  |  | ↑△ |  |  |  |  |  |  | 1 | |
| miR-1290 |  |  |  |  |  |  |  |  |  |  |  |  |  |  |  |  |  |  |  |  |  |  |  |  |  |  |  |  |  | ↑△ |  |  |  |  |  |  | 1 | |
| miR-130a |  |  |  |  |  |  |  |  |  | ↑△ |  |  |  |  |  |  |  |  |  |  |  |  |  |  |  |  |  |  |  |  |  |  |  |  |  |  | 1 | |
| miR-1343-3p |  |  | ↑△ |  |  |  |  |  |  |  |  |  |  |  |  |  |  |  |  |  |  |  |  |  |  |  |  |  |  |  |  |  |  |  |  |  | 1 | |
| miR-141 |  |  |  |  |  |  |  |  |  |  |  |  |  |  |  |  |  |  |  |  |  |  |  |  |  |  |  |  |  |  |  |  | ↑△ |  |  |  | 1 | |
| miR-145-3p |  |  |  |  |  |  |  |  |  |  |  |  | ↑△ |  |  |  |  |  |  |  |  |  |  |  |  |  |  |  |  |  |  |  |  |  |  |  | 1 | |
| miR-150-3p |  |  |  |  |  |  |  |  |  |  |  |  | ↓△ |  |  |  |  |  |  |  |  |  |  |  |  |  |  |  |  |  |  |  |  |  |  |  | 1 | |
| miR-1539 |  |  |  |  |  |  |  |  |  |  |  |  |  |  |  |  |  |  |  |  |  |  |  |  |  |  | ↑○ |  |  |  |  |  |  |  |  |  | 1 | |
| miR-155 |  |  |  |  |  |  |  |  |  |  |  |  | ↓○ |  |  |  |  |  |  |  |  |  |  |  |  |  |  |  |  |  |  |  |  |  |  |  | 1 | |
| miR-15b |  |  |  |  |  |  |  |  |  |  |  |  |  |  |  |  |  |  |  |  |  |  |  |  |  |  |  | ↑△ |  |  |  |  |  |  |  |  | 1 | |
| miR-15b-3p |  |  |  |  |  |  |  |  |  |  |  |  | ↓○ |  |  |  |  |  |  |  |  |  |  |  |  |  |  |  |  |  |  |  |  |  |  |  | 1 | |
| miR-16 |  |  |  |  |  |  |  |  |  |  |  |  |  |  |  |  |  |  |  |  |  |  |  |  |  |  |  | ↑△ |  |  |  |  |  |  |  |  | 1 | |
|  |  |  |  |  |  |  |  |  |  |  |  |  |  |  |  |  |  |  |  |  |  |  |  |  |  |  |  |  |  |  |  |  |  |  |  |  |  | |
| supplementary table 2 continued | | | | | | | | | | | | | | | | | | | | | | | | | | | | | | | | | | | | | | |
| **RNAs** | **(61)** | **(60)** | **(58)** | **(55)** | **(54)** | **(53)** | **(52)** | **(51)** | **(48)** | **(47)** | **(50)** | **(45)** | **(44)** | **(43)** | **(42)** | **(40)** | **(39)** | **(37)** | **(35)** | **(34)** | **(33)** | **(31)** | **(30)** | **(29)** | **(28)** | **(36)** | **(26)** | **(24)** | **(22)** | **(21)** | **(19)** | **(18)** | **(17)** | **(16)** | **(15)** | **(14)** | **Number of**  **studies** | |
| miR-17 |  |  |  |  |  |  |  | ↑△ |  |  |  |  |  |  |  |  |  |  |  |  |  |  |  |  |  |  |  |  |  |  |  |  |  |  |  |  | 1 | |
| miR-181a-2-3p |  |  |  |  |  |  |  |  |  |  |  |  | ↑○ |  |  |  |  |  |  |  |  |  |  |  |  |  |  |  |  |  |  |  |  |  |  |  | 1 | |
| miR-193a-5p |  |  |  |  |  |  |  |  |  |  |  |  |  |  |  |  |  |  |  |  |  | ↓○ |  |  |  |  |  |  |  |  |  |  |  |  |  |  | 1 | |
| miR-186 |  |  |  |  |  |  |  |  |  |  |  |  | ↓○ |  |  |  |  |  |  |  |  |  |  |  |  |  |  |  |  |  |  |  |  |  |  |  | 1 | |
| miR-217 |  |  |  |  | ↓△ |  |  |  |  |  |  |  |  |  |  |  |  |  |  |  |  |  |  |  |  |  |  |  |  |  |  |  |  |  |  |  | 1 | |
| miR-221 |  |  |  |  |  |  |  |  | ↓△ |  |  |  |  |  |  |  |  |  |  |  |  |  |  |  |  |  |  |  |  |  |  |  |  |  |  |  | 1 | |
| miR-222 |  |  |  |  |  |  |  |  |  |  |  |  |  |  |  |  |  |  | ↑○ |  |  |  |  |  |  |  |  |  |  |  |  |  |  |  |  |  | 1 | |
| miR-223 | ↑○ |  |  |  |  |  |  |  |  |  |  |  |  |  |  |  |  |  |  |  |  |  |  |  |  |  |  |  |  |  |  |  |  |  |  |  | 1 | |
| miR-26a |  |  |  |  |  |  |  |  |  |  |  |  | ↓○ |  |  |  |  |  |  |  |  |  |  |  |  |  |  |  |  |  |  |  |  |  |  |  | 1 | |
| miR-26b |  |  |  |  |  |  |  |  |  |  |  |  | ↓○ |  |  |  |  |  |  |  |  |  |  |  |  |  |  |  |  |  |  |  |  |  |  |  | 1 | |
| miR-27 |  |  |  |  |  |  |  |  |  | ↑△ |  |  |  |  |  |  |  |  |  |  |  |  |  |  |  |  |  |  |  |  |  |  |  |  |  |  | 1 | |
| miR-30e |  |  |  |  |  |  |  |  |  |  |  |  | ↓○ |  |  |  |  |  |  |  |  |  |  |  |  |  |  |  |  |  |  |  |  |  |  |  | 1 | |
| miR-31 |  |  |  |  |  |  |  |  |  |  |  |  |  |  |  |  |  |  |  |  |  |  |  |  |  |  |  | ↑△ |  |  |  |  |  |  |  |  | 1 | |
| miR-330-5p |  |  |  |  |  |  |  |  |  |  |  |  | ↑○ |  |  |  |  |  |  |  |  |  |  |  |  |  |  |  |  |  |  |  |  |  |  |  | 1 | |
| miR-339 |  |  |  |  |  |  |  |  |  |  |  |  | ↑○ |  |  |  |  |  |  |  |  |  |  |  |  |  |  |  |  |  |  |  |  |  |  |  | 1 | |
| miR-340 |  |  |  |  |  |  |  |  |  |  |  |  | ↓○ |  |  |  |  |  |  |  |  |  |  |  |  |  |  |  |  |  |  |  |  |  |  |  | 1 | |
| miR-374a-3p |  |  |  |  |  |  |  |  |  |  |  |  | ↓○ |  |  |  |  |  |  |  |  |  |  |  |  |  |  |  |  |  |  |  |  |  |  |  | 1 | |
| miR-377 |  |  |  |  |  |  |  |  |  |  |  |  |  |  |  |  |  |  |  |  |  |  |  |  |  |  |  |  |  |  |  |  |  |  | ↓△ |  | 1 | |
| miR-3937 |  |  |  |  |  |  |  |  |  |  |  |  |  |  |  |  |  |  |  |  |  |  |  |  |  |  |  |  |  |  |  |  |  | ↑○ |  |  | 1 | |
| miR-425-3p |  |  |  |  |  |  |  |  |  |  |  |  | ↑○ |  |  |  |  |  |  |  |  |  |  |  |  |  |  |  |  |  |  |  |  |  |  |  | 1 | |
| miR-484 |  |  |  |  |  |  |  |  |  |  |  |  | ↑○ |  |  |  |  |  |  |  |  |  |  |  |  |  |  |  |  |  |  |  |  |  |  |  | 1 | |
| miR-501-3p |  |  |  |  |  |  |  |  |  |  |  |  | ↑○ |  |  |  |  |  |  |  |  |  |  |  |  |  |  |  |  |  |  |  |  |  |  |  | 1 | |
| miR-543 |  |  | △ |  |  |  |  |  |  |  |  |  |  |  |  |  |  |  |  |  |  |  |  |  |  |  |  |  |  |  |  |  |  |  |  |  | 1 | |
| miR-548c |  |  |  |  |  |  |  |  |  |  |  |  |  |  |  |  |  |  |  |  |  |  |  |  |  |  |  |  |  | -○ |  |  |  |  |  |  | 1 | |
|  |  |  |  |  |  |  |  |  |  |  |  |  |  |  |  |  |  |  |  |  |  |  |  |  |  |  |  |  |  |  |  |  |  |  |  |  |  | |
|  |  |  |  |  |  |  |  |  |  |  |  |  |  |  |  |  |  |  |  |  |  |  |  |  |  |  |  |  |  |  |  |  |  |  |  |  |  | |
| supplementary table 2 continued | | | | | | | | | | | | | | | | | | | | | | | | | | | | | | | | | | | | | | |
| **RNAs** | **(61)** | **(60)** | **(58)** | **(55)** | **(54)** | **(53)** | **(52)** | **(51)** | **(48)** | **(47)** | **(50)** | **(45)** | **(44)** | **(43)** | **(42)** | **(40)** | **(39)** | **(37)** | **(35)** | **(34)** | **(33)** | **(31)** | **(30)** | **(29)** | **(28)** | **(36)** | **(26)** | **(24)** | **(22)** | **(21)** | **(19)** | **(18)** | **(17)** | **(16)** | **(15)** | **(14)** | | **Number of studies** |
| miR-874 |  |  |  |  |  |  |  |  |  |  |  |  |  |  |  |  |  |  |  |  |  |  |  |  | ↓○ |  |  |  |  |  |  |  |  |  |  |  | | 1 |
| miR-576-3p |  |  |  |  |  |  |  |  |  |  |  |  | ↓○ |  |  |  |  |  |  |  |  |  |  |  |  |  |  |  |  |  |  |  |  |  |  |  | | 1 |
| miR-654 |  |  |  |  |  |  |  |  |  |  |  |  |  |  |  |  |  |  |  |  |  |  |  |  |  |  |  |  |  | -○ |  |  |  |  |  |  | | 1 |
| miR-708 |  |  | △ |  |  |  |  |  |  |  |  |  |  |  |  |  |  |  |  |  |  |  |  |  |  |  |  |  |  |  |  |  |  |  |  |  | | 1 |
| miR-92a |  |  |  |  |  |  |  |  |  |  |  |  |  |  |  |  |  |  |  |  |  |  |  |  |  |  |  |  |  |  |  |  | ↑△ |  |  |  | | 1 |
| miR-92b |  |  |  |  |  |  |  |  |  |  |  | ↓○ |  |  |  |  |  |  |  |  |  |  |  |  |  |  |  |  |  |  |  |  |  |  |  |  | | 1 |
| miR-940 |  |  |  |  |  |  |  |  |  |  |  |  |  |  |  |  |  |  |  |  |  |  |  |  |  |  |  |  |  | ↑△ |  |  |  |  |  |  | | 1 |
| miR-99b |  |  |  |  |  |  |  |  |  |  |  |  |  |  |  |  | ↓○ |  |  |  |  |  |  |  |  |  |  |  |  |  |  |  |  |  |  |  | | 1 |
| Let-7a | ↑○ |  |  |  |  |  |  |  |  |  |  |  |  |  |  |  |  |  |  |  |  |  |  |  |  |  |  |  |  |  |  |  |  |  |  |  | | 1 |
| let-7b-3p |  |  |  |  |  |  |  |  |  |  |  |  | ↓△ |  |  |  |  |  |  |  |  |  |  |  |  |  |  |  |  |  |  |  |  |  |  |  | | 1 |
| let-7f-2-3p |  |  |  |  |  |  |  |  |  |  |  |  | ↓○ |  |  |  |  |  |  |  |  |  |  |  |  |  |  |  |  |  |  |  |  |  |  |  | | 1 |
| ∆133p53 |  |  |  |  |  |  |  |  |  |  |  |  |  |  |  |  |  |  |  |  |  |  |  |  |  |  |  |  | ↑○ |  |  |  |  |  |  |  | | 1 |
| ∆Np73 |  |  |  |  |  |  |  |  |  |  |  |  |  |  |  |  |  |  |  |  |  |  |  |  |  |  |  |  | ↑○ |  |  |  |  |  |  |  | | 1 |
| AC008269.1 |  |  |  |  |  |  |  |  |  |  |  |  |  |  |  |  |  |  |  |  |  |  |  |  |  |  |  |  |  |  |  | △ |  |  |  |  | | 1 |
| ANKAR |  |  |  |  |  |  |  |  |  |  |  |  |  |  |  |  |  |  |  |  |  |  |  |  |  |  |  |  |  |  |  | △ |  |  |  |  | | 1 |
| APOL4 |  |  |  |  |  |  |  |  |  |  |  |  |  |  |  |  |  |  |  |  |  |  |  |  |  |  |  |  |  |  |  | △ |  |  |  |  | | 1 |
| BCAR4 |  | △ |  |  |  |  |  |  |  |  |  |  |  |  |  |  |  |  |  |  |  |  |  |  |  |  |  |  |  |  |  |  |  |  |  |  | | 1 |
| CA3 |  |  |  |  |  |  |  |  |  |  |  |  |  |  |  |  |  |  |  |  |  |  |  |  |  |  |  |  |  |  |  | △ |  |  |  |  | | 1 |
| CBWD1 |  |  |  |  |  |  |  |  |  |  |  |  |  |  |  |  |  |  |  |  |  |  |  |  |  |  |  |  |  |  |  | △ |  |  |  |  | | 1 |
| CD133 |  |  |  |  |  |  |  |  |  |  |  |  |  |  |  |  |  |  |  |  |  |  |  |  |  | ↑△ |  |  |  |  |  |  |  |  |  |  | | 1 |
| CD24 |  |  |  |  |  |  |  |  |  |  |  |  |  |  |  |  |  |  |  |  |  |  |  |  |  | ↑△ |  |  |  |  |  |  |  |  |  |  | | 1 |
| CK19 |  |  |  |  |  |  |  |  |  |  |  |  |  |  |  |  |  |  |  |  |  |  |  |  |  | ↑△ |  |  |  |  |  |  |  |  |  |  | | 1 |
| CYP20A1 |  |  |  |  |  |  |  |  |  |  |  |  |  |  |  |  |  |  |  |  |  |  |  |  |  |  |  |  |  |  |  | △ |  |  |  |  | | 1 |
| DMC1 |  |  |  |  |  |  |  |  |  |  |  |  |  |  |  |  |  |  |  |  |  |  |  |  |  |  |  |  |  |  |  | △ |  |  |  |  | | 1 |
| FOXD2-AS1 |  |  |  |  |  |  |  |  |  |  |  |  |  |  |  |  |  |  |  |  |  |  |  |  |  |  |  |  |  |  | ↑△ |  |  |  |  |  | | 1 |
| H19 |  |  |  |  |  |  |  |  |  |  |  |  |  | ↓○ |  |  |  |  |  |  |  |  |  |  |  |  |  |  |  |  |  |  |  |  |  |  | | 1 |
|  |  |  |  |  |  |  |  |  |  |  |  |  |  |  |  |  |  |  |  |  |  |  |  |  |  |  |  |  |  |  |  |  |  |  |  |  | |  |
| supplementary table 2 continued | | | | | | | | | | | | | | | | | | | | | | | | | | | | | | | | | | | | | | |
| **RNAs** | **(61)** | **(60)** | **(58)** | **(55)** | **(54)** | **(53)** | **(52)** | **(51)** | **(48)** | **(47)** | **(50)** | **(45)** | **(44)** | **(43)** | **(42)** | **(40)** | **(39)** | **(37)** | **(35)** | **(34)** | **(33)** | **(31)** | **(30)** | **(29)** | **(28)** | **(36)** | **(26)** | **(24)** | **(22)** | **(21)** | **(19)** | **(18)** | **(17)** | **(16)** | **(15)** | **(14)** | **Number of**  **studies** | |
| HIST2H2AA4 |  |  |  |  |  |  |  |  |  |  |  |  |  |  |  |  |  |  |  |  |  |  |  |  |  |  |  |  |  |  |  | △ |  |  |  |  | 1 | |
| HOTTIP |  |  |  |  |  |  |  |  |  |  |  |  |  | ↓○ |  |  |  |  |  |  |  |  |  |  |  |  |  |  |  |  |  |  |  |  |  |  | 1 | |
| HULC |  |  |  |  |  |  |  |  |  |  |  |  |  | ↓○ |  |  |  |  |  |  |  |  |  |  |  |  |  |  |  |  |  |  |  |  |  |  | 1 | |
| KLHDC8B |  |  |  |  |  |  |  |  |  |  |  |  |  |  |  |  |  |  |  |  |  |  |  |  |  |  |  |  |  |  |  | △ |  |  |  |  | 1 | |
| KRTAP5-4 |  | △ |  |  |  |  |  |  |  |  |  |  |  |  |  |  |  |  |  |  |  |  |  |  |  |  |  |  |  |  |  |  |  |  |  |  | 1 | |
| linc00174 |  |  |  |  |  |  |  |  |  |  |  |  |  |  |  |  |  |  |  |  |  |  |  | ↑○ |  |  |  |  |  |  |  |  |  |  |  |  | 1 | |
| linc01987 |  |  |  |  |  |  |  |  |  |  |  |  |  |  |  |  |  |  |  |  |  |  |  | ↑○ |  |  |  |  |  |  |  |  |  |  |  |  | 1 | |
| linc02037 |  |  |  |  |  |  |  |  |  |  |  |  |  |  |  |  |  |  |  |  |  |  |  | ↑○ |  |  |  |  |  |  |  |  |  |  |  |  | 1 | |
| linc02041 |  |  |  |  |  |  |  |  |  |  |  |  |  |  |  |  |  |  |  |  |  |  |  | ↑○ |  |  |  |  |  |  |  |  |  |  |  |  | 1 | |
| LINC02418 |  |  |  |  |  |  |  |  |  |  |  |  |  |  |  | ↑○ |  |  |  |  |  |  |  |  |  |  |  |  |  |  |  |  |  |  |  |  | 1 | |
| lncRNA XIST |  |  |  |  |  |  |  |  |  |  |  |  |  |  |  |  |  |  |  |  |  |  |  | ↑○ |  |  |  |  |  |  |  |  |  |  |  |  | 1 | |
| LNCV6-108266 |  |  |  |  |  |  |  |  |  |  | ↑○ |  |  |  |  |  |  |  |  |  |  |  |  |  |  |  |  |  |  |  |  |  |  |  |  |  | 1 | |
| LNCV6-116109 |  |  |  |  |  |  |  |  |  |  | ↑○ |  |  |  |  |  |  |  |  |  |  |  |  |  |  |  |  |  |  |  |  |  |  |  |  |  | 1 | |
| LNCV6-38772 |  |  |  |  |  |  |  |  |  |  | ↑○ |  |  |  |  |  |  |  |  |  |  |  |  |  |  |  |  |  |  |  |  |  |  |  |  |  | 1 | |
| LNCV6-84003 |  |  |  |  |  |  |  |  |  |  | ↑○ |  |  |  |  |  |  |  |  |  |  |  |  |  |  |  |  |  |  |  |  |  |  |  |  |  | 1 | |
| LNCV6-98390 |  |  |  |  |  |  |  |  |  |  | ↑○ |  |  |  |  |  |  |  |  |  |  |  |  |  |  |  |  |  |  |  |  |  |  |  |  |  | 1 | |
| LNCV6-98602 |  |  |  |  |  |  |  |  |  |  | ↑○ |  |  |  |  |  |  |  |  |  |  |  |  |  |  |  |  |  |  |  |  |  |  |  |  |  | 1 | |
| MAGEA3 |  | △ |  |  |  |  |  |  |  |  |  |  |  |  |  |  |  |  |  |  |  |  |  |  |  |  |  |  |  |  |  |  |  |  |  |  | 1 | |
| MALAT1 |  |  |  |  |  |  |  |  |  |  |  |  |  | ↓○ |  |  |  |  |  |  |  |  |  |  |  |  |  |  |  |  |  |  |  |  |  |  | 1 | |
| MYC |  |  |  |  |  |  |  |  |  |  |  |  |  |  |  |  |  |  |  |  |  |  |  |  |  | ↑△ |  |  |  |  |  |  |  |  |  |  | 1 | |
| NRIR |  |  |  |  |  |  |  |  |  |  |  |  |  |  |  |  |  |  |  |  |  |  |  |  |  |  |  |  |  |  | ↑△ |  |  |  |  |  | 1 | |
| piR_019825 |  |  | △ |  |  |  |  |  |  |  |  |  |  |  |  |  |  |  |  |  |  |  |  |  |  |  |  |  |  |  |  |  |  |  |  |  | 1 | |
| RAB6D |  |  |  |  |  |  |  |  |  |  |  |  |  |  |  |  |  |  |  |  |  |  |  |  |  |  |  |  |  |  |  | △ |  |  |  |  | 1 | |
| RNA GAS5 |  |  |  |  |  |  |  |  | ↑△ |  |  |  |  |  |  |  |  |  |  |  |  |  |  |  |  |  |  |  |  |  |  |  |  |  |  |  | 1 | |
| SGMS1 |  |  |  |  |  |  |  |  |  |  |  |  |  |  |  |  |  |  |  |  |  |  |  |  |  |  |  |  |  |  |  | △ |  |  |  |  | 1 | |
| STK3 |  |  |  |  |  |  |  |  |  |  |  |  |  |  |  |  |  |  |  |  |  |  |  |  |  |  |  |  |  |  |  | △ |  |  |  |  | 1 | |
|  |  |  |  |  |  |  |  |  |  |  |  |  |  |  |  |  |  |  |  |  |  |  |  |  |  |  |  |  |  |  |  |  |  |  |  |  |  | |
|  |  |  |  |  |  |  |  |  |  |  |  |  |  |  |  |  |  |  |  |  |  |  |  |  |  |  |  |  |  |  |  |  |  |  |  |  |  | |
| supplementary table 2 continued | | | | | | | | | | | | | | | | | | | | | | | | | | | | | | | | | | | | | | |
| **RNAs** | **(61)** | **(60)** | **(58)** | **(55)** | **(54)** | **(53)** | **(52)** | **(51)** | **(48)** | **(47)** | **(50)** | **(45)** | **(44)** | **(43)** | **(42)** | **(40)** | **(39)** | **(37)** | **(35)** | **(34)** | **(33)** | **(31)** | **(30)** | **(29)** | **(28)** | **(36)** | **(26)** | **(24)** | **(22)** | **(21)** | **(19)** | **(18)** | **(17)** | **(16)** | **(15)** | **(14)** | | **Number of studies** |
| H2BFS |  |  |  |  |  |  |  |  |  |  |  |  |  |  |  |  |  |  |  |  |  |  |  |  |  |  |  |  |  |  |  | △ |  |  |  |  | | 1 |
| HIST1H2AI |  |  |  |  |  |  |  |  |  |  |  |  |  |  |  |  |  |  |  |  |  |  |  |  |  |  |  |  |  |  |  | △ |  |  |  |  | | 1 |
| HIST1H2BB |  |  |  |  |  |  |  |  |  |  |  |  |  |  |  |  |  |  |  |  |  |  |  |  |  |  |  |  |  |  |  | △ |  |  |  |  | | 1 |
| TET2-AS1 |  |  |  |  |  |  |  |  |  |  |  |  |  |  |  |  |  |  |  |  |  |  |  | ↑○ |  |  |  |  |  |  |  |  |  |  |  |  | | 1 |
| TUG1 |  |  |  |  |  |  | △ |  |  |  |  |  |  |  |  |  |  |  |  |  |  |  |  |  |  |  |  |  |  |  |  |  |  |  |  |  | | 1 |
| UCA1 |  |  |  |  |  |  | ↑△ |  |  |  |  |  |  |  |  |  |  |  |  |  |  |  |  |  |  |  |  |  |  |  |  |  |  |  |  |  | | 1 |
| UQCRHL |  |  |  |  |  |  |  |  |  |  |  |  |  |  |  |  |  |  |  |  |  |  |  |  |  |  |  |  |  |  |  | △ |  |  |  |  | | 1 |
| VEGF |  |  |  |  |  |  |  |  |  |  |  |  |  |  |  |  |  |  |  |  |  |  |  |  |  | ↑△ |  |  |  |  |  |  |  |  |  |  | | 1 |
| XCL2 |  |  |  |  |  |  |  |  |  |  |  |  |  |  |  |  |  |  |  |  |  |  |  |  |  |  |  |  |  |  |  | △ |  |  |  |  | | 1 |
| XLOC_009459 |  |  |  |  |  |  |  |  |  |  |  |  |  |  |  |  |  |  |  |  |  |  |  |  |  |  |  |  |  |  | ↑△ |  |  |  |  |  | | 1 |
| circ-0004771 |  |  |  |  |  |  |  |  |  |  |  |  |  |  | ↑○ |  |  |  |  |  |  |  |  |  |  |  |  |  |  |  |  |  |  |  |  |  | | 1 |
| circHIPK3 |  |  |  |  |  |  | ↑△ |  |  |  |  |  |  |  |  |  |  |  |  |  |  |  |  |  |  |  |  |  |  |  |  |  |  |  |  |  | | 1 |
| circLPAR1 |  |  |  |  |  |  |  |  |  |  |  |  |  |  |  |  |  |  |  |  |  |  |  |  |  |  |  |  |  |  |  |  |  |  |  | ↓○ | | 1 |
| circ-PNN |  |  |  |  |  |  |  |  |  |  |  |  |  |  |  |  |  |  |  |  |  |  | ↑○ |  |  |  |  |  |  |  |  |  |  |  |  |  | | 1 |
| ○ represents RNAs which have only been analyzed individually and not as part of a miRNA panel; △ represents RNAs which are part of a panel; ↑ represents up-regulation; ↓ represents down-regulation. | | | | | | | | | | | | | | | | | | | | | | | | | | | | | | | | | | | | | | |
