# Supplementary material for: Extracellular vesicle biomarkers in circulation for colorectal cancer detection: a systematic review and meta-analysis
Source: BMC Cancer. 2024 May 22;24:623. doi: 10.1186/s12885-024-12312-8 (PMC11110411; doi:10.1186/s12885-024-12312-8)
Supplement: Supplementary file 10 — Supplementary Material 10 [file 12885_2024_12312_MOESM10_ESM.docx]

| **Supplementary table 6** Summary of studies reporting significant associations of proteins in colorectal cancer | | | | | | | | | | | | | |
| --- | --- | --- | --- | --- | --- | --- | --- | --- | --- | --- | --- | --- | --- |
| **Proteins** | **(59)** | **(57)** | **(56)** | **(49)** | **(46)** | **(41)** | **(38)** | **(32)** | **(27)** | **(25)** | **(20)** | **(23)** | **Number of studies** |
| CD63 |  |  |  | △ |  |  |  | △ |  |  |  | △ | 3 |
| EpCAM | △ | △ |  |  |  |  |  | △ |  |  |  |  | 3 |
| CD147 | △ |  |  |  | ○ |  |  |  |  |  |  |  | 2 |
| CD9 |  |  |  |  |  |  |  | △ |  |  |  | △ | 2 |
| ANGPT1 |  |  | △ |  |  |  |  |  |  |  |  |  | 1 |
| ANXA11 |  |  | ○ |  |  |  |  |  |  |  |  |  | 1 |
| ANXA3 |  |  | ○ |  |  |  |  |  |  |  |  |  | 1 |
| ANXA4 |  |  | ○ |  |  |  |  |  |  |  |  |  | 1 |
| ANXA5 |  |  | ○ |  |  |  |  |  |  |  |  |  | 1 |
| C9 |  |  | ○ |  |  |  |  |  |  |  |  |  | 1 |
| CD88 |  |  | ○ |  |  |  |  |  |  |  |  |  | 1 |
| CEACAM8 |  |  | ○ |  |  |  |  |  |  |  |  |  | 1 |
| CLRN3 |  |  |  |  |  |  |  |  |  |  |  | △ | 1 |
| CPNE3 |  |  |  |  |  | ○ |  |  |  |  |  |  | 1 |
| EGFR |  | △ |  |  |  |  |  |  |  |  |  |  | 1 |
| EMMPRIN |  | △ |  |  |  |  |  |  |  |  |  |  | 1 |
| FGA |  |  |  |  |  |  |  |  | ○ |  |  |  | 1 |
| FGB |  |  |  |  |  |  |  |  |  |  | △ |  | 1 |
| FITC |  |  |  |  |  |  |  |  |  |  |  | △ | 1 |
|  |  |  |  |  |  |  |  |  |  |  |  |  |  |
| supplementary table 3 continued | | | | | | | | | | | | | |
| **Proteins** | **(59)** | **(57)** | **(56)** | **(49)** | **(46)** | **(41)** | **(38)** | **(32)** | **(27)** | **(25)** | **(20)** | **(23)** | **Number of studies** |
| GCNT3 |  |  |  |  |  |  |  |  |  |  |  | △ | 1 |
| GLUT-1 |  |  | △ |  |  |  |  |  |  |  |  |  | 1 |
| GRB2 |  |  | ○ |  |  |  |  |  |  |  |  |  | 1 |
| HSPA2 |  |  | ○ |  |  |  |  |  |  |  |  |  | 1 |
| HSPA5 |  |  | △ |  |  |  |  |  |  |  |  |  | 1 |
| LCN2 |  |  | △ |  |  |  |  |  |  |  |  |  | 1 |
| LRG1 |  |  |  |  |  |  | ○ |  |  |  |  |  | 1 |
| MEP1A |  |  |  |  |  |  |  |  |  |  |  | △ | 1 |
| MMP9 |  |  | △ |  |  |  |  |  |  |  |  |  | 1 |
| MUC1 |  | △ |  |  |  |  |  |  |  |  |  |  | 1 |
| MUC5B |  |  | ○ |  |  |  |  |  |  |  |  |  | 1 |
| Mucin12 |  |  |  |  |  |  |  |  |  |  |  | △ | 1 |
| OLFM4 |  |  | △ |  |  |  |  |  |  |  |  |  | 1 |
| ORM1 |  |  | ○ |  |  |  |  |  |  |  |  |  | 1 |
| PerCP |  |  |  |  |  |  |  |  |  |  |  | △ | 1 |
| PIGY |  |  |  |  |  |  |  |  |  |  |  | △ | 1 |
| PSMA5 |  |  | ○ |  |  |  |  |  |  |  |  |  | 1 |
| QSOX1 |  |  |  |  |  |  |  |  |  | ○ |  |  | 1 |
| REG4 |  |  |  |  |  |  |  |  |  |  |  | △ | 1 |
|  |  |  |  |  |  |  |  |  |  |  |  |  |  |
| supplementary table 3 continued | | | | | | | | | | | | | |
| **Proteins** | **(59)** | **(57)** | **(56)** | **(49)** | **(46)** | **(41)** | **(38)** | **(32)** | **(27)** | **(25)** | **(20)** | **(23)** | **Number of studies** |
| SLC1A5 |  |  | ○ |  |  |  |  |  |  |  |  |  | 1 |
| SPARC |  |  |  |  |  |  | ○ |  |  |  |  |  | 1 |
| TFRC |  |  | △ |  |  |  |  |  |  |  |  |  | 1 |
| TNN |  |  | ○ |  |  |  |  |  |  |  |  |  | 1 |
| TPP1 |  |  | △ |  |  |  |  |  |  |  |  |  | 1 |
| TSPAN1 |  |  |  | △ |  |  |  |  |  |  |  |  | 1 |
| β2-GP1 |  |  |  |  |  |  |  |  |  |  | △ |  | 1 |
| ○ represents proteins which have only been analyzed individually and not as part of a protein panel; △ represents proteins which are part of a panel. | | | | | | | | | | | | | |
